# A specific role of iron in promoting meristematic cell division during adventitious root formation

**DOI:** 10.1093/jxb/erx248

**Published:** 2017-08-07

**Authors:** Alexander Hilo, Fahimeh Shahinnia, Uwe Druege, Philipp Franken, Michael Melzer, Twan Rutten, Nicolaus von Wirén, Mohammad-Reza Hajirezaei

**Affiliations:** 1 Leibniz Institute of Plant Genetics and Crop Plant Research, Corrensstraße, Gatersleben, Germany; 2 Leibniz Institute of Vegetable and Ornamental Crops, Kuehnhaeuser Straße, Erfurt, Germany

**Keywords:** Adventitious root formation, iron, meristem, organ formation, plant nutrition, root development

## Abstract

Adventitious root (AR) formation is characterized by a sequence of physiological and morphological processes and determined by external factors, including mineral nutrition, the impacts of which remain largely elusive. Morphological and anatomical evaluation of the effects of mineral elements on AR formation in leafy cuttings of *Petunia hybrida* revealed a striking stimulation by iron (Fe) and a promotive action of ammonium (NH_4_^+^). The optimal application period for these nutrients corresponded to early division of meristematic cells in the rooting zone and coincided with increased transcript levels of mitotic cyclins. Fe-localization studies revealed an enhanced allocation of Fe to the nuclei of meristematic cells in AR initials. NH_4_^+^ supply promoted AR formation to a lesser extent, most likely by favoring the availability of Fe. We conclude that Fe acts locally by promoting cell division in the meristematic cells of AR primordia. These results highlight a specific biological function of Fe in AR development and point to an unexploited importance of Fe for the vegetative propagation of plants from cuttings.

## Teaser

The essential role of iron in formation of adventitious roots is associated with an enhanced allocation of this element to actively dividing cells, highlighting a specific function in meristem development.

## Introduction

In horticulture, agriculture and forestry, adventitious root (AR) formation is a prerequisite for the vegetative propagation of many important crops and common practice to maintain genetic identity in progeny (Hartmann *et al.*, 2011). In spite of optimized conditioning of environmental and production factors in the modern propagation industry, insufficient rooting still results in economic losses. A deeper understanding of the physiological and molecular mechanisms of AR formation is therefore essential for the improvement of existing propagation protocols.

The *de novo* formation of ARs is a complex developmental process during which the stem base of cuttings undergoes a series of anatomical and physiological transformations. Traditionally, phases of AR formation are classified into (i) induction, (ii) initiation of ARs, and (iii) expression (Kevers *et al.*, 1997; Li *et al.*, 2009). Induction of founder cells occurs before any histological changes become apparent and is associated with the physical separation of the cutting from the stock plant. Disruption of polar auxin transport caused by severance of the cutting leads to the accumulation of indole-3-acetic acid in its stem base, which is considered to be a prerequisite for induction of the founder cells (De Klerk *et al.*, 1999; Garrido *et al.*, 2002; Pop *et al.*, 2011; Ahkami *et al.*, 2013). Furthermore, disturbance of existing cell connections and transport of assimilates causes a depletion of assimilates that in turn leads to the establishment of a new carbohydrate sink in the stem base (Ahkami *et al.*, 2009).

The initiation phase is characterized by intense divisions of founder cells leading to the formation of meristems and organization of AR primordia that further protrude through the stem during the expression phase (Kevers *et al.*, 1997). These two phases are characterized by an increase in the assimilate supply towards the rooting zone, necessary for the division and differentiation of the meristematic cells (Veierskov *et al.*, 1982; Druege *et al.*, 2004; Ahkami *et al.*, 2009).

In addition to metabolic rearrangements, nutrient supply to the stem base of the cuttings could also affect the efficiency of AR formation. In this regard, the nutritional status of the stock plants may play an important role in providing an endogenous pool of nutrients required to support AR formation at the stem base. For this reason, the role of mineral nutrition in AR formation has been studied mainly with respect to the fertilization of the stock plants. Indeed, a high initial N content in cuttings of *Pelargonium* × *hortorum* Bailey and *Euphorbia pulcherrima* Willd. has been shown to exert a positive effect on AR formation (Druege *et al.*, 2000; Zerche & Druege, 2009). However, so far, little attention has been paid to the role of the rooting medium as a potential source of essential nutrients. Empirical trials with *in vitro* propagated plant material provided evidence for the importance of the mineral composition of the medium in successful formation of ARs (Murashige, 1974). The pioneering study by Schwambach *et al.* (2005) highlighted the importance of balanced nutrient supply at specific stages of AR formation in microcuttings of *Eucalyptus globulus* Labill. For instance, optimized supply levels of Ca, Zn and NO_3_^−^ to the medium increased the number of ARs. In another study, Santos & Fisher (2009) showed that application of an NPK fertilizer directly to the stem base of petunia (*Petunia hybrida* Vilm.) cuttings during root emergence improved rooting, whereas foliar application of nutrients had no effect on root development.

In Arabidopsis (*Arabidopsis thaliana* (L.) Heynh.), studies on lateral root (LR) formation emphasized the ability of plants to alter their root system architecture in response to the availability of certain nutrients. Localized exposure of a part of the root system to nitrate (NO_3_^−^), P, Zn or Fe has been shown to favor LR elongation, whereas the localized supply of ammonium (NH_4_^+^) stimulated higher order lateral root branching (Zhang and Forde, 1998; Linkohr *et al.*, 2002; Lima *et al.*, 2010; Giehl *et al.*, 2012). Although the molecular mechanisms underlying the local sensing of specific nutrients remain to be fully elucidated, several lines of evidence suggest that phytohormones, and in particular auxin, act as signals in the nutrient-dependent modification of root morphology (Giehl *et al.*, 2014). In fact, auxin has been shown to regulate lateral root elongation in response to localized NO_3_^−^ and Fe application, even though the defined role of auxin appears to differ (Krouk *et al.*, 2010; Giehl *et al.*, 2012). Partial resemblance of auxin-mediated signaling pathways controlling the induction and the development of LRs and ARs (da Costa *et al.*, 2013; Bellini *et al.*, 2014) suggests that regulatory networks involved in mineral sensing by LRs can be employed for the stimulation of AR development.

Petunia is a highly important ornamental plant in world-wide horticulture with established biochemical and molecular platforms that make it a convenient model plant for the characterization of AR formation (Ahkami *et al.*, 2009, 2014; Druege *et al.*, 2014). Transcriptome analysis of AR formation in petunia revealed a substantial increase in the expression of 18 genes involved in the uptake and assimilation of N, P, K, S, Fe and Zn starting from the initiation phase (Ahkami *et al.*, 2014). Moreover, within this period, high transcript abundance was observed for a plasma membrane H^+^-ATPase, which may energize nutrient uptake. These findings emphasize the increased demand for certain mineral elements in the stem base and suggest that AR formation in petunia cuttings may be improved by application of certain nutrients.

Therefore a primary aim of this study was to characterize the role of targeted nutrient supplies in the stimulation of AR formation in leafy cuttings of petunia. Nutrient application may affect different physiological processes, including compensation of nutrient deficiency, modulation of carbon metabolism in the basal stem zone, or changes in the balance of phytohormones. Here, we combined morphological, anatomical, biochemical and molecular approaches in order to characterize the influence of selected nutrients throughout AR formation and to investigate their modes of action.

## Materials and methods

### Plant material, growth conditions and sampling

Leafy stem cuttings of *Petunia hybrida* cv. Mitchell were used for all experiments. Stock plants were grown in the glasshouse at 22 °C and approximately 85% relative humidity during 10 h of insolation (250 µmol m^−2^ s^−1^), and 20 °C and 60% relative humidity during 14 h of darkness. Excised leafy cuttings with four to five leaves were transferred to a hydroponic system with permanent aeration under equal light and temperature conditions. The full mineral solution contained 0.1 mM KH_2_PO_4_, 0.1 mM MgSO_4_, 0.25 mM CaCl_2_, 2 mM NH_4_NO_3_, 0.01 mM Fe-ethylenediamine-*N*,*N*′-bis(2-hydroxyphenylacetic acid) (FeEDDHA), 0.050 mM Н_З_ВО_З_, 0.005 mM MnSO_4_, 0.001 mM ZnSO_4_, 0.001 mM CuSO_4_, 0.0007 mM NaMoO_4_ and 1 mM MES, pH 5.8, in distilled water. The influence of individual nutrients was studied in buffered solution (control conditions) consisting of 1 mM CaSO_4_ and 1.5 mM MES (pH 5.8). Ammonium and nitrate were supplied as (NH_4_)_2_SO_4_ and Ca(NO_3_)_2_, respectively. Iron was supplied as FeEDDHA. Hydroponic solutions were replaced every 5 d. For the split-shoot experiment, a 2-cm incision was made through the middle of the stem of the cutting, and each half of the stem base was placed in a separate well of a culture plate (96-DeepWell, Nunc). Cultivation was performed in hydroponic solutions, which were replaced daily.

Samples from mature leaves and 5 mm of each cutting base were harvested 6, 24, 72, 120 and 168 h after excision, immediately frozen in liquid nitrogen and stored at −80 °C.

### Morphological and anatomical assessment of AR formation

Two days after appearance of the first ARs under control conditions, which occurred 12–14 d post excision (dpe), the roots of each cutting (three replicates, ten cuttings each) were counted and assigned to different length classes of 5-mm intervals. The percentage of rooted cuttings, the average number of roots per rooted cutting and the average root length were determined as described in Agulló-Antón *et al.* (2011).

For histological examination 1-mm thick cross-sections of petunia stem cuttings were subjected to combined conventional and microwave-assisted fixation, dehydration and resin embedding in a microwave processor (PELCO BioWave34700-230, Ted Pella) as described in Supplementary Table S1 at *JXB* online. Semi-thin sectioning and light microscopy were performed according to Ahkami *et al.* (2013).

For analysis of the dynamics of AR development, 100-µm thick segments of the cutting base were made at 1 mm intervals with a vibrating blade microtome (VT-1000S, Leica Microsystems) and prepared as described in Supplementary Table S2 for analysis using a digital microscope (VHX-5000, Keyence). AR initials were divided into five classes: I, meristemoids; II, globular meristems; III, AR primordia with dome-shaped meristems; IV, AR primordia with elongated cells and developing vasculature; V, emerged AR. The average number of AR initials in each category was calculated for groups of 12 independent replicates 5, 6, 7, and 9 dpe in control cuttings and cuttings supplied with NH_4_^+^, NO_3_^−^, or Fe.

Preparation of the samples for the quantification of AR primordia in the split-shoot experiment was carried out essentially as described in Supplementary Table S2 except that samples were fixed and bleached in FAA solution (2% (v/v) formaldehyde, 5% (v/v) acetic acid in 70% (v/v) ethanol) followed by rehydration in a series of decreasing ethanol concentrations that allowed omitting the subsequent staining of sections.

### Histochemical detection of Fe

For visualization of Fe deposition, stem base specimens were fixed as described in Supplementary Table S2, dehydrated in a graded ethanol series, and embedded in wax as described by Braszewska-Zalewska *et al.* (2013). The histochemical detection of Fe was carried out using Perls’ Prussian blue staining with 3,3′-diaminobenzidine (DAB)/H_2_O_2_ intensification, according to Roschzttardtz *et al.* (2009). Light microscopy analysis was performed using a digital microscope (VHX-5000, Keyence). For visualization of nuclei, Perls’ Prussian blue–DAB-stained samples were incubated with 0.2 mg l^−1^ of 4′,6-diamidino-2-phenylindole (DAPI). Photospectrometric analysis of sections was performed using a laser scanning microscope (LSM780, Carl Zeiss) with excitation at 405 nm (1.8% intensity); emission was measured over the range 411–482 nm, after which DAPI-specific fluorescence corresponding to 461 nm was unmixed. Bright-field recordings were taken with a 633-nm laser line using dark-field settings.

### Measurement of mineral elements and chlorophyll

To analyse major macro- and micronutrients, freeze-dried material was digested in HNO_3_ under pressure using a microwave digester (Ultraclave-4; Milestone). Elemental analysis was performed using a sector field high-resolution inductively coupled plasma mass spectrometer (ELEMENT-2, Thermo Fisher Scientific). Total N and C were determined using an elemental analyser (Euro-EA; HEKAtech). The concentrations of chlorophyll *a* and *b* were analysed in methanol extracts of leaf samples according to Warren (2008). Absorbance at 652 and 665 nm was measured using a microplate reader (Infinite-200, Tecan).

### Targeted metabolite profiling

Carbohydrates, primary intermediates of sugar metabolism and amino acids were extracted and analysed essentially as described by Höller *et al.* (2014), except that the separation of amino acids was carried out by UPLC (AcQuity H-Class, Waters) on a C18 reversed-phase column (ACCQ-Tag UltraC18, 1.7 µM, 2.1 × 100 mm) at 50 °C, with a flow rate of 0.7 ml min^−1^, run time of 10.2 min, excitation at 266 nm and detection at 473 nm. Detailed settings for tandem mass spectrometry analysis of metabolites are provided in Supplementary Table S3.

### Gene expression analysis

Total RNA was isolated from the stem bases of at least eight cuttings per experimental group, according to Logemann *et al.* (1987), followed by treatment with DNAse-I (Qiagen) and synthesis of first-strand cDNA using M-MLV reverse transcriptase (Promega) according to the manufacturer’s protocol. Gene expression was analysed by real-time RT-qPCR (CFX384, Bio-Rad) using iQ™-SYBRGreen Supermix (Bio-Rad). Gene-specific primers were designed to have a melting temperature of 58 °C and to result in a PCR product of between 150 and 250 bp (see Supplementary Table S4). Relative transcript levels of time-course values were determined by the ${\text{2}}_{}^{-\Delta \Delta C_{\text{t}}}$ method and related to the initial transcript level 0 dpe. The level of *ACTIN7* mRNA was selected as a suitable internal reference based on previous studies (Ahkami *et al.*, 2013, 2014) and its showing stable expression under all studied conditions throughout the course of experiment (see Supplementary Fig. S1). All assays were performed on three biological and two technical replicates. Each analysis was repeated at least twice.

### Generation of DR5::GFP-GUS petunia reporter line

A binary vector p9N-DR5-GFP-GUSi (see Supplementary Fig. S2) containing *EGFP-GUS* fusion with *KDEL* and *LeB4SP* retention signal peptides, derived from the pGH183 vector (kindly provided by Dr Goetz Hensel, IPK, Gatersleben), an auxin-inducible synthetic promoter DR5 from the pS001:DR5-GFP vector (Friml *et al.*, 2003) and a backbone of A560p9Ndoi-TOCS vector (DNA-Cloning Service Hamburg) were constructed and mobilized into *Agrobacterium tumefaciens* GV2260 according to Sambrook *et al.* (1989). Transformation of *Petunia hybrida* cv. Mitchell leaf explants was carried out according to Lutke (2006), and regenerated shoots were selected on 100 mg l^−1^ kanamycin.

### Statistical analyses

Analysis of variance (ANOVA) provided by InfoStat^®^ software was used for statistical analysis of data. In case of significant impact of the factor, Fisher’s LSD or Tukey’s HSD were conducted at *P*≤0.05. Comparison between the treatments and controls was carried out by use of Student’s *t*-test at *P*≤0.05.

## Results

### Determination of mineral nutrients essential for AR formation

**Fig. 1.**
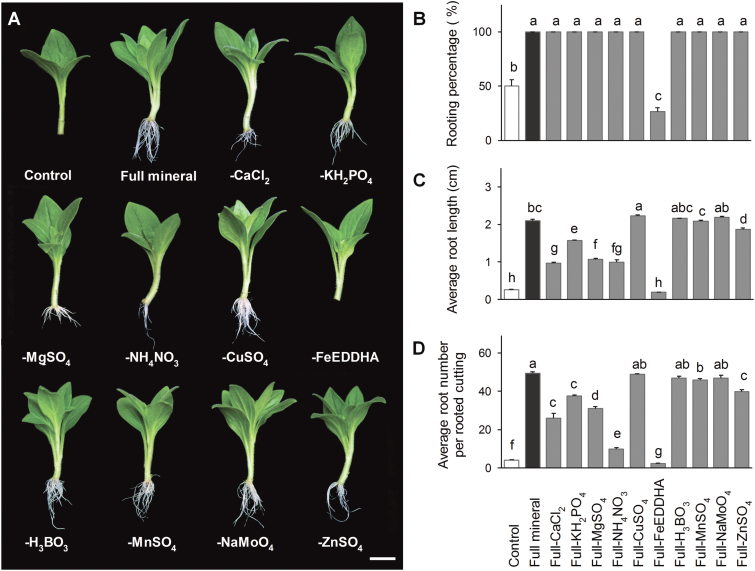
Effect of the withdrawal of components from full mineral solution on adventitious root (AR) formation in *Petunia hybrida*. (A) Representative image of rooted cuttings in nutrient-free solution (control), full mineral solution or full mineral solution lacking the indicated nutrients. (B–D) Percentage of rooted cuttings (B), average root length (C), and average number of ARs (D) were each assessed 14 d after excision. Bars represent means+SE of three independent replicates each consisting of ten cuttings. Significant differences are indicated by different letters (Fisher’s LSD, *P*≤0.05). Scale bar, 1 cm.

In leafy cuttings of petunia, *P.* × *hortorum* and *Dendranthema grandiflorum* Kitam., rooting was frequently observed in inert media without any external nutrient supply (Druege *et al.*, 2000, 2004; Ahkami *et al.*, 2009), indicating that the cuttings themselves contain sufficient amounts of nutrients to develop ARs. In the present study, cultivation in full mineral medium significantly improved rooting (Fig. 1), emphasizing the importance and potential role of externally supplied nutrients in AR formation. To first determine which nutrients limit AR formation, individual components were excluded from the full mineral medium (Fig. 1). Withdrawal of CuSO_4_, H_3_BO_3_, MnSO_4_, or NaMoO_4_ appeared to have no significant effect on rooting. The average number and the length of ARs decreased significantly in cuttings deprived of KH_2_PO_4_, MgSO_4_, CaCl_2_, and ZnSO_4_, but all cuttings still rooted. Omitting NH_4_NO_3_ decreased the average root length eight-fold and the average root number by five-fold compared with the full medium (Fig. 1C, D), which pointed to a potential role of nitrogen in AR formation. An even greater negative effect on rooting performance was observed upon withdrawal of Fe. This resulted in little or no rooting, similar to the control conditions, suggesting that the major effect of the full nutrient supply in promoting AR formation was due to the presence of Fe (Fig. 1). Based on these results, we focused our further investigations on the influence of Fe and N on AR formation.

**Fig. 2.**
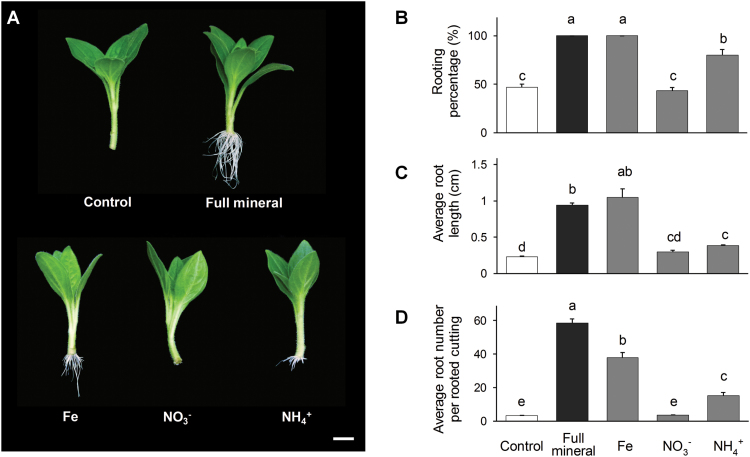
Effect of the application of individual elements on adventitious root (AR) formation in *Petunia hybrida*. (A) Representative image of rooted cuttings supplied with iron, nitrate, or ammonium compared with nutrient-free medium or full mineral supply. (B–D) Percentage of rooted cuttings (B), average root length (C), and average number of ARs (D) were each assessed 14 d after excision. Bars represent means+SE of three independent replicates, each consisting of ten cuttings. Significant differences are indicated by different letters (Fisher’s LSD, *P*≤0.05). Scale bar, 1 cm.

Initial experiments identified that the optimum concentration of Fe leading to a significant increase in rooting parameters was between 8 and 12 µM (see Supplementary Table S5). The application of 10 µM Fe alone had a positive effect on AR formation, resulting in 100% rooting, which occurred 2–3 d earlier compared with the control solution (Fig. 2). The average root length increased three-fold, to a similar level as in full mineral medium, whereas the average number of ARs increased ten-fold compared with the control (Fig. 2C, D). The positive effect on the rooting performance of N supplied as NH_4_NO_3_ was most pronounced at a concentration of 0.2 mM (Supplementary Table S5). In order to distinguish between the influence of different N forms, NO_3_^−^ and NH_4_^+^ were supplied separately at a concentration of 0.4 mM. Approximately 80% of NH_4_^+^-supplied cuttings developed ARs 14 dpe, compared with 43.3% and 46.7% under NO_3_^−^ application or control conditions, respectively (Fig. 2D). The average number of ARs increased three-fold in NH_4_^+^-supplied cuttings, whereas NO_3_^−^ application resulted in values similar to control conditions.

**Fig. 3.**
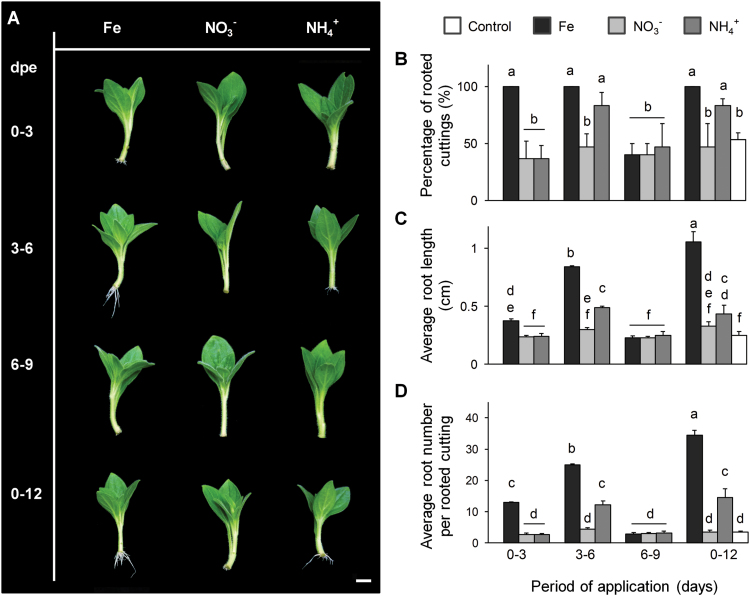
Effect of a short-term application of individual nutrients on adventitious root (AR) formation in *Petunia hybrida*. (A) Representative image of rooted cuttings in response to a supply of nutrients for a separate period of 3 d compared with continuous application of nutrients. (B–D) Percentage of rooted cuttings (B), average root length (C), and average number of ARs (D) were each assessed 14 d after excision. Bars represent means+SE of three independent replicates, each consisting of ten cuttings. Significant differences are indicated by different letters (Tukey’s HSD, *P*≤0.05). Scale bar, 1 cm.

To determine the critical developmental stage for nutrient application, Fe, NO_3_^−^, or NH_4_^+^ was supplied for separate periods of 3 d during the first 9 dpe (Fig. 3). Application of Fe during 0–3 and 3–6 dpe significantly improved the rooting, with the highest number and length of ARs being detected in cuttings supplied during 3–6 dpe. As before, AR formation under NO_3_^−^ application remained poor. Application of NH_4_^+^ only for the period of 3–6 dpe improved the rooting performance to a similar level as observed in cuttings supplied with NH_4_^+^ during the entire period of cultivation. Thus, the developmental period between 3 and 6 dpe appeared to be the most sensitive for stimulation of AR formation.

### Anatomy of AR formation in response to nutrient application

**Fig. 4.**
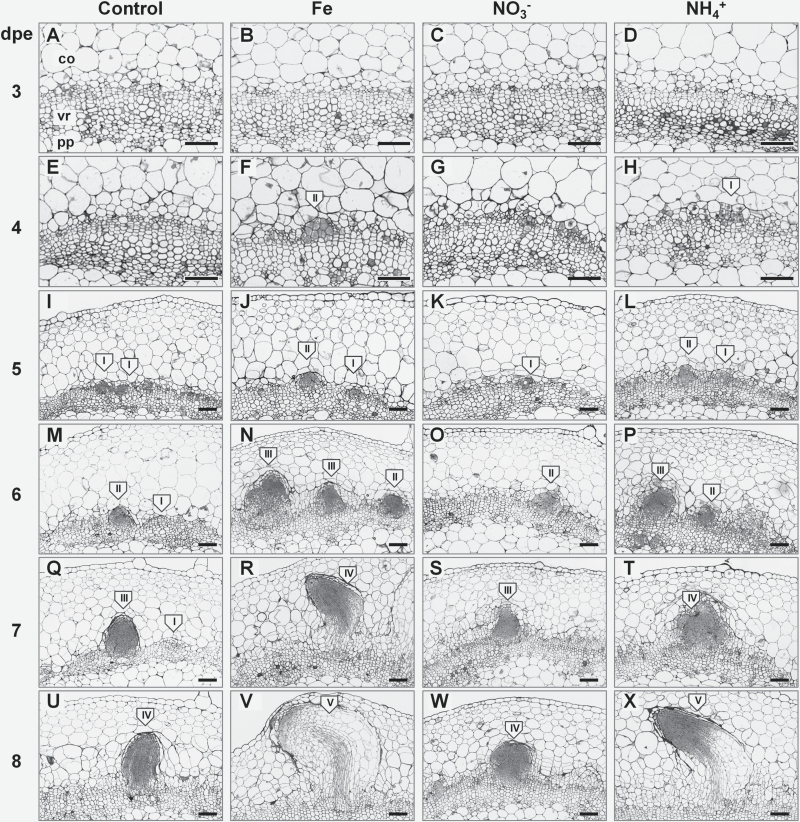
Anatomy of adventitious root (AR) formation of *Petunia hybrida* cuttings in response to nutrient application. Cross sections from 1–4 mm of the stem base are shown 3–8 dpe in cuttings grown without nutrients or separately with iron, nitrate, or ammonium. Developmental stages of AR initials are indicated in roman numbers: I, meristemoids; II, globular meristems; III, AR primordia with dome-shaped meristems; IV, AR primordia with elongated cells and developing vasculature; V, emerged AR. co, cortex; pp, pith parenchyma; vr, vascular ring. Scale bar, 200 µm.

In order to investigate whether promotion of AR formation by Fe and NH_4_^+^ application is accompanied by histological changes in the stem base, we performed a structural analysis using light microscopy. In this study, the first meristemoids, composed of meristematic cells with dense cytoplasm and a large nucleus, were observed 4 dpe in the rooting zone of the cuttings supplied with Fe or NH_4_^+^ (Fig. 4F, H). Under control and NO_3_^−^ conditions only single dividing cells were detected (Fig 4E, G). Differences among nutrient applications became more distinct following the progress of AR primordia formation. In the cuttings supplied with Fe or NH_4_^+^ the first globular AR meristemoids were observed 5 dpe (Fig. 4J, L), and early AR primordia with dome-shaped meristems were detected 6 dpe (Fig. 4N, P), whereas in control and NO_3_^−^-supplied cuttings similar formations appeared 1 d later (Fig. 4M, O, Q, S). The first AR primordia with defined elongation zones and developing vasculature were observed 7 dpe in Fe- or NH_4_^+^-supplied cuttings (Fig. 4R, T) but again 1 d later in control or NO_3_^−^-supplied cuttings (Fig. 4U, W). The first protrusion of ARs out of the stem cortex occurred 8 dpe in Fe- or NH_4_^+^-treated cuttings (Fig. 4V, X).

**Fig. 5.**
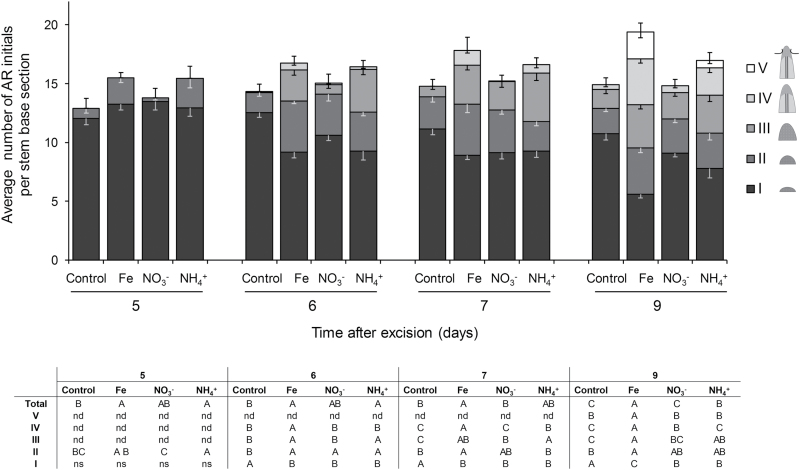
Quantitative assessment of the dynamics of adventitious root (AR) formation in *Petunia hybrida*. Developing AR initials were divided into 5 classes: I, meristemoids; II, globular meristems; III, AR primordia with dome-shaped meristems; IV, AR primordia with elongated cells and developing vasculature; V, emerged AR. The average number of each of the defined AR initials in 100 µm cross-sections of the rooting zone is shown 5, 6, 7 and 9 dpe at three different levels in cuttings grown without nutrients, or with ammonium, nitrate, or iron. Bars represent means−SE of 12 independent replicates. The total number of AR initials is represented by the size of the stacked columns +SE. The table shows by different letters significant differences in the number of specific AR initials in given classes for each time point (Fisher’s LSD, *P*≤0.05). nd, structures not detected; ns, difference is not significant.

To assess the dynamics of AR development, we recorded the numbers of AR initials at the stages defined above during 5–9 dpe (Fig. 5). The number of total detectable AR initials 5 dpe was higher in the rooting zone of the cuttings supplied with Fe or NH_4_^+^. Relative to control conditions, application of NO_3_^−^ resulted in a similar total number of AR initials 9 dpe, whereas the highest values were observed in the cuttings supplied with Fe, and, to a lesser extent, NH_4_^+^.

The number of meristemoids (Fig. 5) was comparable in all conditions 5 dpe, followed by a significant decrease at later stages, observed especially upon application of Fe and, to a lesser degree, NH_4_^+^, which indicated accelerated progression through the subsequent stages of AR primordia development in response to these nutrients.

### Mineral element status during AR formation in response to nutrient application

**Fig. 6.**
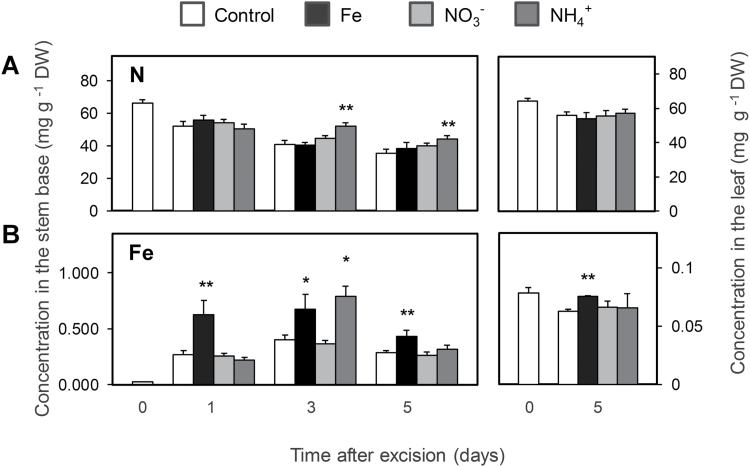
Concentrations of nitrogen (A) and iron (B) in the stem base and in mature leaves of *Petunia hybrida* cuttings during adventitious root formation in response to nutrient application. Bars represent means+SE of five independent cuttings. Significant differences from control treatments at specified time points after excision are indicated by asterisks (*t*-test, *P<0.05, **P<0.01).

Element analysis revealed a gradual decline in the concentration of N in the stem base throughout the period of measurements (Fig. 6A). NH_4_^+^-supplied cuttings maintained a significantly higher level of total N compared with both the control and NO_3_^−^ application. In mature leaves the level of N was not affected by nutrient application (Fig. 6A).

The concentration of Fe in the stem base of the control cuttings had increased ten-fold by 1 dpe, whereas application of Fe resulted in a 30-fold increase 1 and 3 dpe (Fig. 6B). The stem base of NH_4_^+^-supplied cuttings accumulated significantly higher levels of Fe 3 dpe. In mature leaves, Fe concentrations decreased 5 dpe in the control, while they remained stable under Fe supply. This indicated that an enhanced N level in the stem base was unlikely to explain the improved AR formation, whereas enhanced Fe levels as observed after supply of either Fe or NH_4_^+^ coincided with improved AR formation.

### Effect of localized Fe application on AR formation

**Fig. 7.**
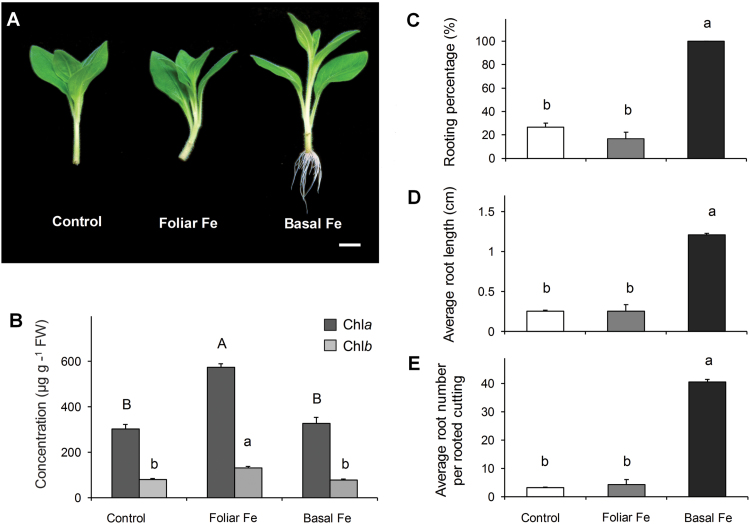
Effect of foliar or basal application of iron on adventitious root (AR) formation in *Petunia hybrida*. (A) Representative image of the rooted cuttings in response to foliar or basal application of Fe compared with control conditions. (B) Chlorophyll *a* or *b* concentrations in mature leaves shown as the mean+SE of five independent replicates, analysed 14 dpe. (C–E) Percentage of rooted cuttings (C), average root length (D), and average number of ARs (E) were assessed 14 d after excision. Bars represent means+SE of three independent replicates, each consisting of ten cuttings. Significant differences are indicated by different letters (Fisher’s LSD, *P*≤0.05). Scale bar, 200 µm.

In order to distinguish whether the positive effect of Fe application on AR formation is achieved by maintenance of its level in the leaves or performance of a specific function in the rooting zone, foliar application of Fe was compared with application of Fe to the rooting medium. Although foliar application of Fe significantly increased leaf chlorophyll concentrations as compared with Fe supplied via the nutrient solution (Fig. 7B), the rooting performance of leaf-supplied cuttings remained at a similar low level to that observed under nutrient-free conditions. It is clear that AR formation was improved only by application of Fe to the cutting base (Fig. 7C–E).

**Fig. 8.**
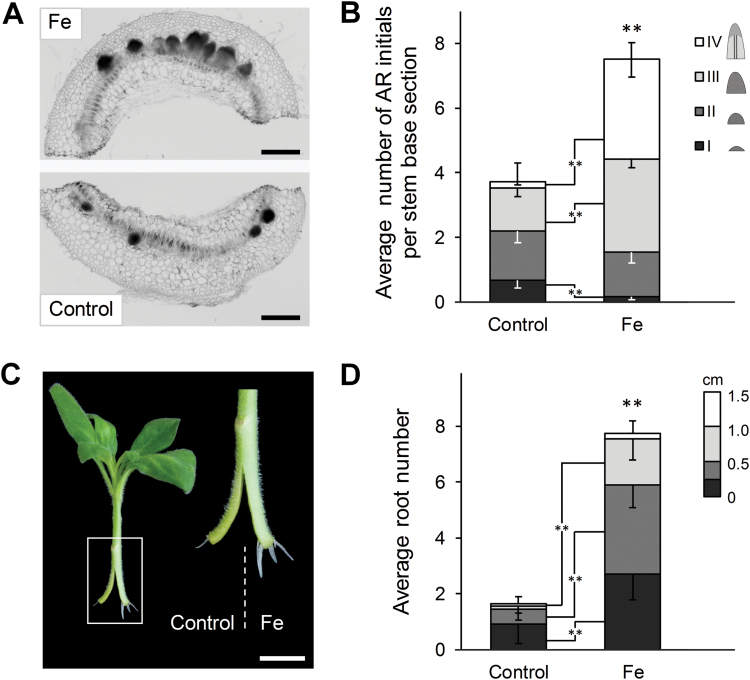
Effect of local iron supply on adventitious root (AR) formation in split-shoot cuttings of *Petunia hybrida.* Shoot cuttings were split at the stem base and one half was grown without nutrients (control), while the other was grown in presence of 10 µM Fe-EDTA. (A) Representative image of a cross-section through the rooting zone of the split-shoot cutting 6 d post excision (dpe). Scale bar, 500 µm. (B) Quantitative assessment of AR formation 6 dpe. Developing AR initials in 100 µm cross-sections of the rooting zone were divided into 4 classes: I, meristemoids; II, globular meristems; III, AR primordia with dome-shaped meristems; IV, AR primordia with elongated cells and developing vasculature. Bars represent means−SE of each of the defined AR initial classes from 16 independent replicates. The total number of AR initials is represented by the size of the stacked columns +SE. Significant differences from control treatments in the number of specific AR initials in a given class are indicated by asterisks (*t*-test, **P<0.01). (C) Representative image of the rooted cutting 14 dpe. (D) Average number of ARs 14 dpe. Bars represent means−SE of each of the defined AR length classes from 24 independent replicates. The total number of ARs is represented by the size of the stacked columns +SE. Significant differences from control treatments in the number of specific AR initials in a given length class are indicated by asterisks (*t*-test, **P<0.01). Scale bar, 1 cm.

To further confirm the local effect of Fe, AR formation was analysed in a split-shoot experiment. Two parts of a radially cut stem base of a cutting were differentially supplied with Fe (Fig. 8). Assessment of the numbers of AR initials 6 dpe indicated that the number of detectable AR initials was two-fold higher in the Fe-supplied part of the cutting base (Fig. 8A, B). Moreover, the Fe-supplied part of the stem was characterized by a higher number of AR primordia in a more advanced stage of development (6 dpe) and a significantly higher number of protruded ARs 12 dpe (Fig. 8C, D). Thus, improved AR formation strongly relies on a local rather than a systemic action of available Fe.

### Impact of nutrient application on the level of primary N and C metabolites

Among the analysed free amino acids, the level of glutamine and asparagine in the stem base decreased significantly 3 dpe, followed by a recovery 5 dpe to the initial values (see Supplementary Fig. S3A, C). However, in cuttings supplied with NH_4_^+^ the level of glutamine and asparagine increased approximately two-fold from 3 dpe with a simultaneous decrease in the concentrations of glutamic and aspartic acid (Supplementary Fig. S3B, D). In mature leaves the levels of the analysed amino acids were not affected by nutrient application. Concentrations of soluble carbohydrates, as well as concentrations of primary intermediates of sugar metabolism, showed no significant differences in response to the nutrient supply (Supplementary Figs S3E, F and S4), indicating that the nutrient-dependent AR formation at this stage is not primarily associated with an enhanced accumulation of primary metabolites in the stem base of the cutting.

### Effect of auxin regulators on AR formation in response to nutrient application

As several studies report the involvement of auxin in LR and AR formation (Sánchez *et al.*, 2007; Swarup *et al.*, 2008; Pop *et al.*, 2011; Gutierrez *et al.*, 2012; Della Rovere *et al.*, 2013) we treated nutrient-supplied cuttings with *N*-1-naphthylphthalamic acid (NPA) or L-kynurenine (Kyn) as inhibitors of polar transport or *de novo* biosynthesis of auxin, respectively. Application of NPA negatively affected rooting performance under all of the studied conditions (see Supplementary Fig. S5). In control and NO_3_^−^ conditions NPA decreased rooting percentage by almost three-fold with a significant reduction of the average length of ARs compared with non-treated cuttings. Rooting percentage and average AR number of NH_4_^+^-supplied cuttings declined by approximately 60% in response to NPA along with a more than two-fold reduction in average AR length. In contrast to other nutrient applications, all of the Fe-supplied cuttings treated with NPA developed AR. However, the average number and length of ARs decreased by more than 40% compared with non-treated cuttings.

Treatment with Kyn showed negative effects on rooting performance similar to those of NPA under control and NO_3_^−^ conditions, whereas rooting percentage and average AR number of NH_4_^+^-supplied cuttings decreased by approximately 60% (see Supplementary Fig. S5). Application of Kyn to Fe-supplied cuttings had no significant effect on the rooting percentage and average AR length, while the average number of ARs decreased by 15% compared with the non-treated cuttings. Thus, Fe-dependent AR formation was much less affected by inhibition of auxin biosynthesis than NH_4_^+^-dependent AR formation.

In order to distinguish between the effect of auxin and Fe on AR formation, rooting performance was assessed in cuttings supplied with Fe or nutrient-free medium in combination with 1-naphthaleneacetic acid (NAA) during the induction phase (0–2 dpe; Supplementary Fig. S6). In cuttings deprived of nutrients and treated with NAA, the rooting percentage reached maximum, whereas the average AR number increased by approximately 90% compared with untreated cuttings, to a similar number as under sole Fe supply (see Supplementary Fig. S6B, D). Fe-supplied cuttings developed approximately 40% more ARs following NAA treatment (Supplementary Fig. S6D). As auxin acted on top of Fe-mediated AR formation in petunia cuttings, both treatments contributed in an additive manner.

### Impact of iron on the activity of the auxin reporter DR5::GFP-GUS

To additionally investigate the spatial distribution of the auxin response during Fe-dependent AR formation, we generated an auxin reporter line by expressing a green fluorescent protein (GFP)–β-glucuronidase (GUS) fusion construct under control of the auxin-inducible DR5 promoter. Within 1 dpe, the GFP-specific fluorescence under Fe supply or nutrient-free conditions was primarily associated with the inner and outer phloem rings as well as being adjacent to cortical cells (see Supplementary Fig. S7A, B). At 3 dpe, intense fluorescence appeared as bright spots on both sides of the vessel ring, corresponding to dividing meristematic cells of the developing AR primordia, which showed similar fluorescence intensity despite being more advanced under Fe supply (Supplementary Fig. S7C, D). Following 7 dpe, the maximum DR5 promoter activity under either condition was concentrated at the tip and in the vasculature of AR primordia (Supplementary Fig. S7E, F). Therefore, despite anatomical differences in primordia development, observed changes in the distribution of auxin response appeared to be independent of Fe supply.

Furthermore, to confirm the observations with DR5 promoter activity, the associated enzyme activity of the GUS–GFP fusion protein was analysed (see Supplementary Fig. S8). In this assay, an almost linear increase in GUS activity was recorded with progressing AR formation, although no significant difference in GUS activity in response to nutrient application was detected. The observed continuous increase in activity was explained by the high stability of the GUS protein against plant proteases, leading to accumulation of the enzyme even if auxin maintained a constant level in developing AR.

### Influence of nutrients on transcript abundance of marker genes for cell division, nutrient acquisition and auxin homeostasis

**Fig. 9.**
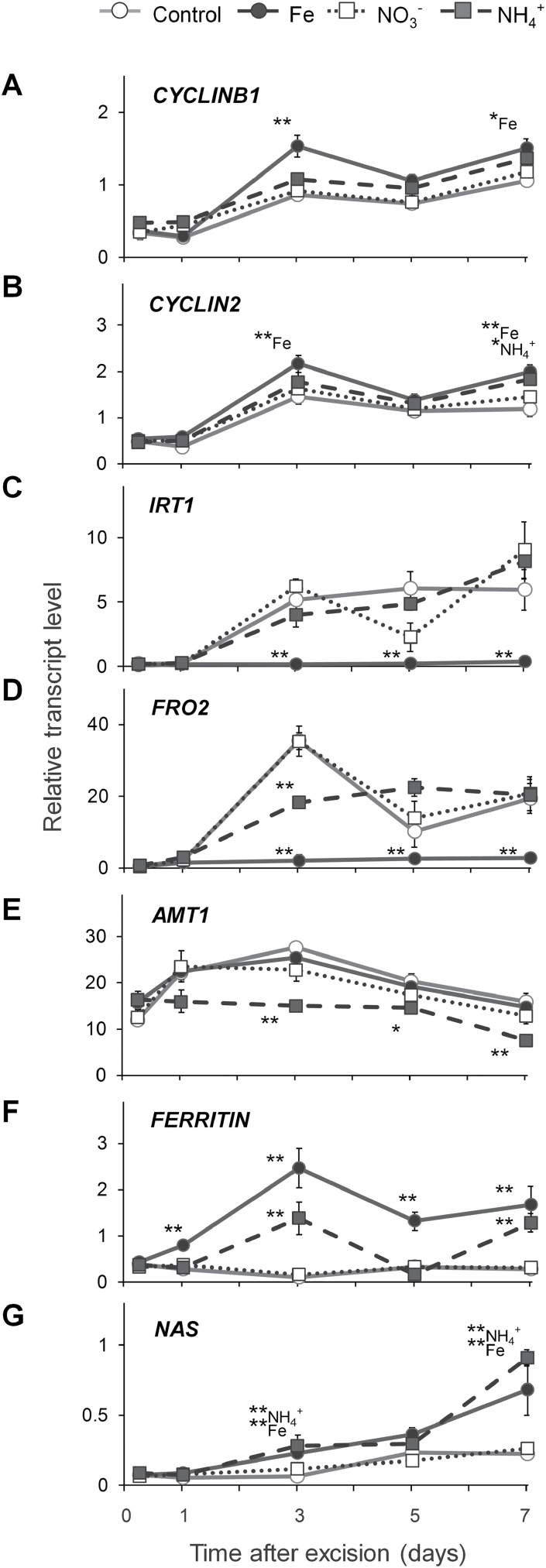
Transcript abundance of key genes in the stem base of *Petunia hybrida* cuttings during adventitious root formation in response to nutrient application. (A) *CYCLINB1*, (B) *CYCLIN2*, (C) *IRT1*, (D) *FRO2*, (E) *AMT1*, (F) *FERRITIN*, (G) *NAS*. Each data point represents the mean±SE of three independent biological replicates of the fold-change related to the initial transcript level at the time of excision. Significant differences to control treatments at specified time points after excision are indicated by asterisks (t-test, *P<0.05, **P<0.01).

Transcript abundance of the *CYCLINB1* gene has been suggested as a marker for mitotic activity (Ferreira *et al.*, 1994; de Almeida Engler *et al.*, 1999). In agreement with the study by Ahkami *et al.* (2009), *CYCLINB1* transcript levels increased 3 dpe and were significantly higher in the stem base of Fe-supplied cuttings 3 and 7 dpe (Fig. 9A). A similar pattern of transcript accumulation was detected for the G2/mitotic-specific marker *CYCLIN2* with significantly higher values 7 dpe under NH_4_^+^ supply and 3 and 7 dpe under Fe supply (Fig. 9B).

As dicotyledonous plant, petunia acquires Fe via the strategy I mechanism, which involves the reduction of Fe^III^ to Fe^II^ by the ferric-chelate reductase FRO2 and subsequent Fe^2+^ uptake by IRT1 (Vert *et al.*, 2002). Under control conditions, mRNA levels of petunia *IRT1* homolog increased by five-fold from 3 dpe onwards, whereas in Fe-supplied samples transcripts remained at a basal level (Fig. 9C). Similar patterns of transcript accumulation were recorded for *FRO2* (Fig. 9D).

Under control conditions, transcript levels of the N-responsive NH_4_^+^ transporter gene *AMT1* (Gazzarrini *et al.*, 1999; Vert *et al.*, 2002) showed a 20-fold increase from 1 dpe, peaking 3 dpe, followed by a gradual decrease (Fig. 9E). Application of NO_3_^−^ did not affect transcript levels of *AMT1*, whereas NH_4_^+^ application led to a significant decrease throughout the course of analysis.

Transcript levels of the Fe storage protein ferritin, *FERRITIN*, were significantly downregulated in control and NO_3_^−^-supplied cuttings from 6 dpe, whereas Fe application led to a significant increase from 1 dpe, reaching a peak 3 dpe (Fig. 9F). Interestingly, transcript accumulation after NH_4_^+^ application was less pronounced but resembled that observed in Fe-supplied cuttings. Transcript levels of nicotianamine synthase, *NAS*, responsible for synthesis of the intracellular Fe chelator nicotianamine (von Wiren *et al.*, 1999), showed an immediate ten-fold decrease after excision in all of the studied conditions, followed by a significantly higher accumulation 3 and 7 dpe in the case of Fe or NH_4_^+^ supply (Fig. 9G).

### Effect of the medium pH on AR formation

Based on the fact that NH_4_^+^ nutrition leads to physiological acidification of the rooting environment (Taylor and Bloom, 1998), the effect of medium pH on AR development was examined (Supplementary Fig. S9). Despite the absence of NH_4_^+^ as an N source, all of the rooting parameters were significantly increased when cuttings were cultivated at pH 4.5 as compared with pH 5.8 in control conditions. In contrast, a shift towards more alkaline conditions resulted in a significant decrease of the rooting performance. Thus, a drop in apoplast or rhizosphere pH, as typically observed under NH_4_^+^ nutrition, promotes AR formation, either directly as consequence of cell wall acidification or indirectly via improved Fe acquisition.

### Cellular localization of iron during AR formation

**Fig. 10.**
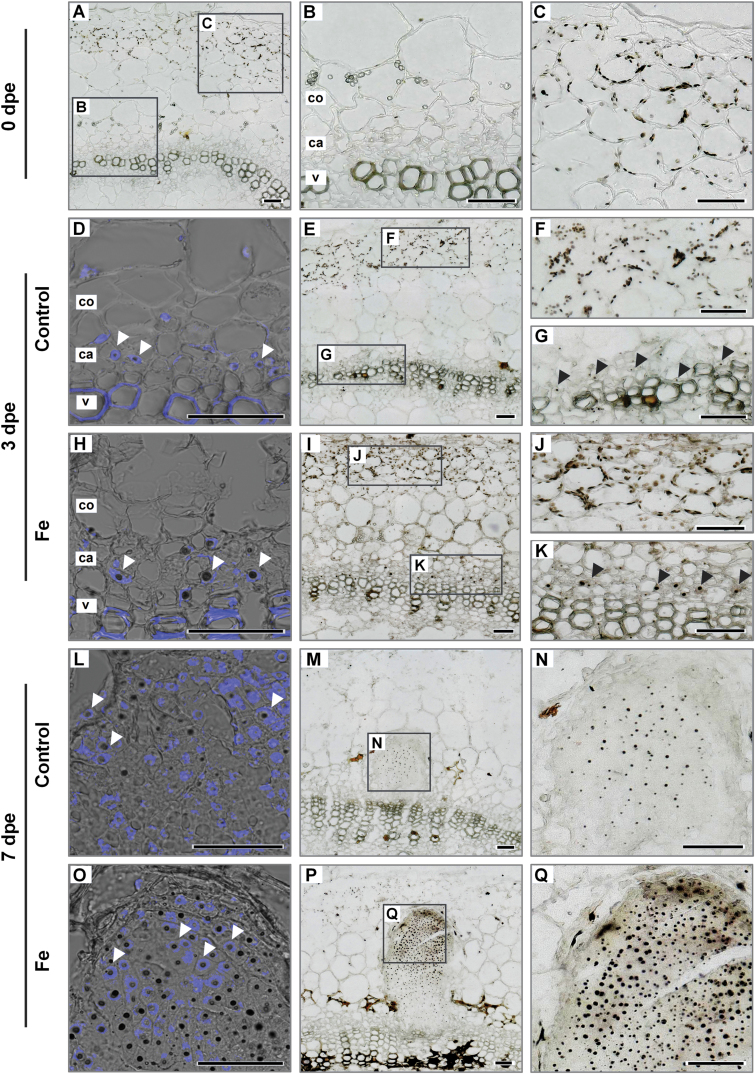
Localization of Fe by Perls’ Prussian blue–DAB method in the stem base of *Petunia hybrida* cuttings in response to Fe supply during adventitious root formation. Cross sections from 1–4 mm of the stem base are shown 0 dpe (A–C), 3 dpe (D–K) and 7 dpe (L–Q) in cuttings grown without nutrients (A–G; L–N) or with Fe (H–K; O–Q). Black arrowheads show Fe localization in cambial cells 3 dpe. Additional staining with DAPI (D, H, L, O) indicates nuclear localized Fe in meristematic cells (white arrowheads). ca. cambium; co. cortex; pp. pith parenchyma; v. vessels of xylem. Scale bar, 50 µm.

As Fe application increased transcript levels of cyclins (Fig. 9A, B), we hypothesized that Fe may stimulate cell cycle-related processes in the nucleus. We therefore assessed the dynamics of local Fe distribution during AR primordia formation using Perls’ Prussian blue–DAB. Immediately after excision (0 dpe), Fe was stained inside cortical cells in oval structures located proximal to the surface of the stem (Fig. 10). These Fe-containing structures most likely represented chloroplasts of the photosynthetically active stem tissue (Fig. 10A, C). In control conditions 3 dpe, small black dots of stained Fe appeared also in the center of cells adjacent to the vessel ring, corresponding to the cambium and outer phloem (Fig. 10E, G). Under Fe supply, the intensity of stained Fe increased in the chloroplasts of cortical cells and even more in the cambium and outer phloem. The intensity of Fe staining in cortical cells 7 dpe decreased considerably under both conditions with a relatively paler staining observed in control cuttings (Fig. 10M, P). However, the most striking difference in Fe allocation was detected in developing AR primordia. While under control conditions Fe was only detected in the form of small spots in some of the primordial cells (Fig. 10N), primordial cells of Fe-supplied cuttings were more intensely stained with a gradient of Fe accumulation towards the primordial apex (Fig. 10Q). The Fe-containing spots were visibly larger than under Fe-free conditions and increased in size with proximity to the apex of AR primordia (Fig. 10Q).

To verify whether the Fe-accumulating intracellular compartment corresponded to the nucleus, DAPI staining was combined with the Perls’ Prussian blue–DAB method. Indeed, Fe in the phloem cells 3 dpe and in meristematic cells of AR primordia 7 dpe co-localized with nuclear-specific DAPI fluorescence (Fig. 10D, H, L, O). These results indicated that Fe is specifically allocated to the nuclei of meristematic cells from the early initiation events onwards.

## Discussion

The progress of AR formation is associated with several physiological bottlenecks, among which the impact of mineral nutrition has remained largely elusive. On the assumption that the application of any of the essential mineral elements should improve rooting performance, AR formation was assessed in the presence of different micronutrients or forms of N in order to investigate the impact of these nutrients on AR formation. Unexpectedly, this study revealed a highly stimulating role of Fe and a promotive action of NH_4_^+^ on AR formation. In addition to immediate benefits for horticultural application, the present study highlights the biological action of these two nutrients in the development of AR.

### Promotion of AR formation by iron and ammonium is related neither to the shoot nutritional status nor to auxin levels in the stem base

Previous studies have indicated the accumulation of several nutrients, including Cu, Fe and Mg, in the stem base of *Pinus taeda* L. and *E. pulcherrima* cuttings during the course of AR formation (Svenson and Davies, 1995; Rowe *et al.*, 1999). Moreover, application of combined NPK fertilizer at specific stages improved rooting in petunia cuttings (Santos and Fisher, 2009). Our systematic approach by omitting individual nutrients from the nutrient solution demonstrated that AR formation in leafy cuttings of petunia does not improve with the general nutritional status of the plant. Instead, only two nutrients were found to confer beneficial effects: NH_4_^+^, and in particular Fe. Mineral element analysis revealed that, even in the absence of Fe, petunia cuttings accumulated this element in the stem base already after 1 d (Fig. 6B) suggesting that Fe retranslocation may have taken place. Although foliar Fe application apparently met the Fe demand of the cutting and prevented a drop of chlorophyll levels in leaves, none of the rooting parameters improved (Fig. 7C–E). This suggested that Fe takes over a specific role in the rooting zone during AR formation, which cannot be achieved by shoot-to-root translocation of leaf Fe, and that the Fe demand relevant for AR formation depends on a local provision of Fe to the stem base rather than on the systemic Fe nutritional status of the stem cutting.

Evidence for a specific role of N during AR formation came from studies in *D. grandiflorum* and *E. pulcherrima*, which showed that the number and length of ARs positively correlated with the initial N concentration in the cuttings (Druege *et al.*, 2000; Zerche & Druege, 2009). In the present study only NH_4_^+^-supplied cuttings (and not those supplemented with NO_3_^−^) had a higher concentration of total N (Fig. 6A), which was reflected by elevated concentrations of glutamine and asparagine (Supplementary Fig. S3A, C). These results indicated a more rapid assimilation of NH_4_^+^ directly in the stem base, which in turn may have decreased transcript levels of N-regulated genes like the ammonium transporter *AMT1* (Fig. 9E). However, in all treatments N levels remained above 4%, which reflects an adequate N nutritional status (Hawkesford *et al.*, 2012). Thus, shoot N accumulation *per se* was most likely not a trigger for AR development in NH_4_^+^-supplied cuttings, suggesting that indirect effects may play a role, such as nutrient-dependent alterations in phytohormone homeostasis.

Auxin acts at multiple levels in the initiation and development of LRs and ARs, and is also involved in LR elongation in response to local NO_3_^−^ and Fe availability (Krouk *et al.*, 2010; Kiba *et al.*, 2011; Giehl *et al.*, 2012). The present study also supports a role of auxin in AR development, since the combined effect of auxin inhibitors together with nutrients indicated that stimulation of AR development by Fe and NH_4_^+^ requires auxin and involves polar auxin transport (see Supplementary Fig. S5). However, evidence from this study indicates that nutrient-dependent promotion of AR formation acted independently from auxin in the stem base: (i) the spatial distribution and activity of the auxin-reporter GFP–GUS was not affected by Fe supply despite striking differences in the rate of primordia development (Supplementary Figs S7 and S8), and (ii) the additive effect on the enhancement of rooting under combined application of Fe and NAA suggests parallel mechanisms of stimulation of AR formation by Fe application and auxin (Supplementary Fig. S6).

### A local function of iron in the early development of AR primordia

**Fig. 11.**
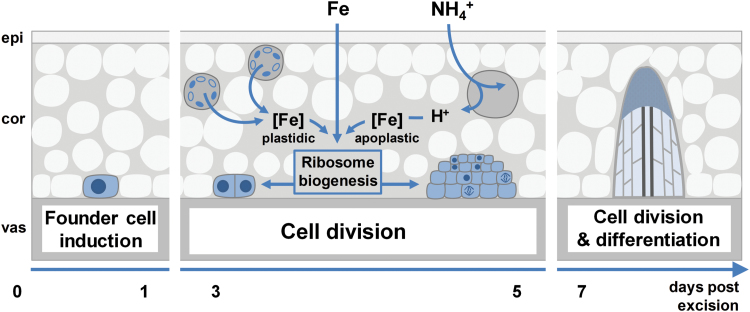
Working model for the effects of iron and ammonium on adventitious root (AR) formation in *Petunia hybrida*. Fe performs a specific function in the rooting zone by activating ribosome biogenensis, crucial for growth of the meristematic cells of the developing AR. Under Fe-free conditions Fe associated with chloroplasts in cortical cell layers may be released to supply developing AR meristems. The uptake of NH_4_^+^ leads to a local acidification of the apoplast as a consequence of an increased net H^+^ efflux, which in turn facilitates the mobilization of Fe precipitated in the apoplast. cor, cortex; epi, epidermis; vas, vasculature. (This figure is available in color at *JXB* online.)

The present investigations in petunia highlight that Fe application enhances the growth of ARs by stimulating the division of early meristematic cells as well as by accelerating progression of AR initials through the different developmental stages (Figs 4, 5, and 8A, B). Among the possible functions of Fe in AR formation, involvement in the stimulation of cell division of meristematic cells appears most plausible. Studies in animals and yeast have highlighted a role of Fe in maintenance of DNA stability and in control of the cell cycle (Zhang, 2014). Moreover, Roschzttardtz *et al.* (2011) identified a surprisingly high accumulation of Fe in the nucleolus of plant cells, suggesting that this element may be involved in ribosomal RNA biosynthesis. Indeed, the rate of ribosome biogenesis appears to correlate with cell proliferation (Manzano *et al.*, 2013), which becomes a crucial factor in actively dividing meristematic cells. In this study, external application of Fe stimulated AR formation most efficiently within 3–6 dpe (Fig. 3), which coincided with increased transcript levels of mitotic cyclins (Fig. 9A, B). In addition, only the Fe-supplied part of the split-shoot cutting was characterized by enhanced AR formation (Fig. 8). Therefore, we propose that a local action of Fe on meristematic cells is required for enhanced cell division (Fig. 11). Such a view is supported by Fe localization studies, showing a strongly enhanced allocation of Fe towards the early dividing cells 3 dpe as well as to apical meristems of AR primordia 7 dpe (Fig. 10). Moreover, in the absence of an external supply, Fe disappeared from chloroplasts in cortical cell layers (Fig. 10), indicating that Fe may be released from degrading chloroplasts, whenever the stem base is not exposed to light and photosynthesis declines.

### Ammonium facilitates iron-mediated AR formation

Several lines of evidence indicated that NH_4_^+^ supply promoted AR formation via similar mechanisms as suggested for Fe: (i) the phase-specific application of NH_4_^+^ promoted rooting percentage, average root length, and average root number within the same time window as Fe supply (Fig. 3); (ii) as with Fe, NH_4_^+^ produced meristemoids 1 d earlier than under control conditions or with NO_3_^−^ (Fig. 4), and accelerated progression of AR initials through different developmental stages (Fig. 5); (iii) like Fe, also NH_4_^+^ stimulated transcript abundance of the *CYCLIN2* as well as the two Fe-related genes *FERRITIN* and *NAS* (Fig. 9F, G); and (iv) NH_4_^+^ supply increased Fe concentrations in the stem base 3 dpe to a similar extent to Fe supply (Fig. 6). In all these cases, NH_4_^+^ supply conferred a similar but weaker stimulation compared with Fe supply, resulting in qualitatively similar dynamics of AR development to that in Fe-supplied cuttings (Fig. 5). We therefore propose that NH_4_^+^ supply leads to a local improvement of Fe nutrition at the stem base (Fig. 11). NH_4_^+^ uptake is known to cause physiological acidification due to an enhanced net H^+^ efflux (Taylor and Bloom, 1998). Even in buffered solutions, apoplastic pH may locally decrease and facilitate solubilization of Fe previously precipitated in the cell walls. Such a scenario is in line with the observation that 3 dpe NH_4_^+^ supply increased transcript levels of *FERRITIN* (Fig. 9F), suggesting that the shoot Fe nutritional status improved. A primarily pH-triggered action of NH_4_^+^ finds support in a separate experiment, in which low pH enhanced AR formation (see Supplementary Fig. S9). By contrast, in most experiments NO_3_^−^ supply led to similar AR formation to that in untreated control plants, which is in agreement with its physiological alkalinization of the apoplast and thus the lack of an Fe-solubilizing effect.

Taken together, the present study shows an unprecedented stimulation of AR development in petunia cuttings by local Fe supply. Thereby, Fe accelerates the development of AR meristemoids. This effect is partly mimicked by supply of NH_4_^+^, which most likely improves Fe availability at the stem base by its acidifying action. Implementing these findings in current protocols used in horticulture for the propagation of petunia and possibly other related species has great potential for improving rooting success and thus decreasing losses.

## Supplementary data

Supplementary data are available at *JXB* online.

Fig. S1. Analysis of transcript abundance of *ACTIN7* and *EF1α* in this study.

Fig. S2. The map of the binary vector p9N-DR5-GFP-GUSi.

Fig. S3. Concentrations of major amino acids and carbohydrates in petunia cuttings.

Fig. S4. Concentrations of primary sugar metabolites in petunia cuttings.

Fig. S5. Effect of auxin inhibitors on AR formation in petunia cuttings.

Fig. S6. Effect of NAA application on AR formation in petunia cuttings.

Fig. S7. GFP fluorescence in the stem base of DR5::GUS/GFP auxin-reporter line.

Fig. S8. GUS-activity in the stem base of DR5::GUS/GFP auxin-reporter line.

Fig. S9. Effect of the medium pH on AR formation in petunia cuttings.

Table S1. Protocol for fixation and resin embedding of samples from petunia cuttings.

Table S2. Protocol for histological preparation of samples from petunia cuttings.

Table S3. Settings for MS/MS analysis of primary metabolites.

Table S4. Primers used in this study.

Table S5. Effect of Fe or N supply on AR formation in petunia cuttings.

## Author contributions

AH designed and performed the experiments, evaluated the data and wrote the manuscript. FS established the hydroponic system and performed the initial experiments. UD and PF were throughout involved in the study from formulating hypotheses up to improving the paper. MM and TR performed and evaluated the experiments for light microscopy. NvW revised and strongly improved the quality of the paper. MRH conceived the project, designed and supervised the experiments and revised the manuscript.

## Supplementary Material

Supplementary_Figures_S1_S9_Table_S1_S5

## Abbreviations:

AR: adventitious root
DAB: 3,3′-diaminobenzidine
DAPI: 4′,6-diamidino-2-phenylindole
Kyn: L-kynurenine
LR: lateral root
NAA: 1-naphthaleneacetic acid
NPA: *N*-1-naphthylphthalamic acid.
