# Supplementary material for: A specific role of iron in promoting meristematic cell division during adventitious root formation
Source: J Exp Bot. 2017 Aug 7;68(15):4233–47. doi: 10.1093/jxb/erx248 (PMC5853222; doi:10.1093/jxb/erx248)
Supplement: Supplementary_Figures_S1_S9_Table_S1_S5 [file erx248_suppl_supplementary_figures_s1_s9_table_s1_s5.pdf]

A specific role of iron in promoting meristematic cell division during adventitious root formation

Alexander Hilo, Fahimeh Shahinnia, Uwe Druege, Philipp Franken, Michael Melzer, Twan Rutten, Nicolaus von Wirén and Mohammad-Reza Hajirezaei

Supplementary data

Supplementary data are available at JXB online.

Figure S1 Analysis of transcript abundance of *ACTIN7* and *EF1α* in this study.
Figure S2 Map of the binary vector p9N-DR5-GFP-GUSi.
Figure S3 Concentrations of major amino acids and carbohydrates in petunia cuttings.
Figure S4 Concentrations of primary sugar metabolites in petunia cuttings.
Figure S5 Effect of auxin inhibitors on AR formation in petunia cuttings.
Figure S6 Effect of NAA application on AR formation in petunia cuttings.
Figure S7 GFP fluorescence in the stem base of DR5::GUS/GFP auxin-reporter line.
Figure S8 GUS-activity in the stem base of DR5::GUS/GFP auxin-reporter line.
Figure S9 Effect of the medium pH on AR formation in petunia cuttings.
Table S1 Protocol for fixation and resin embedding of samples from petunia cuttings.
Table S2 Protocol for histological preparation of samples from petunia cuttings.
Table S3 Settings for MS/MS analysis of primary metabolites
Table S4 Primers used in this study.
Table S5 Effect of Fe or N supply on AR formation in petunia cuttings.

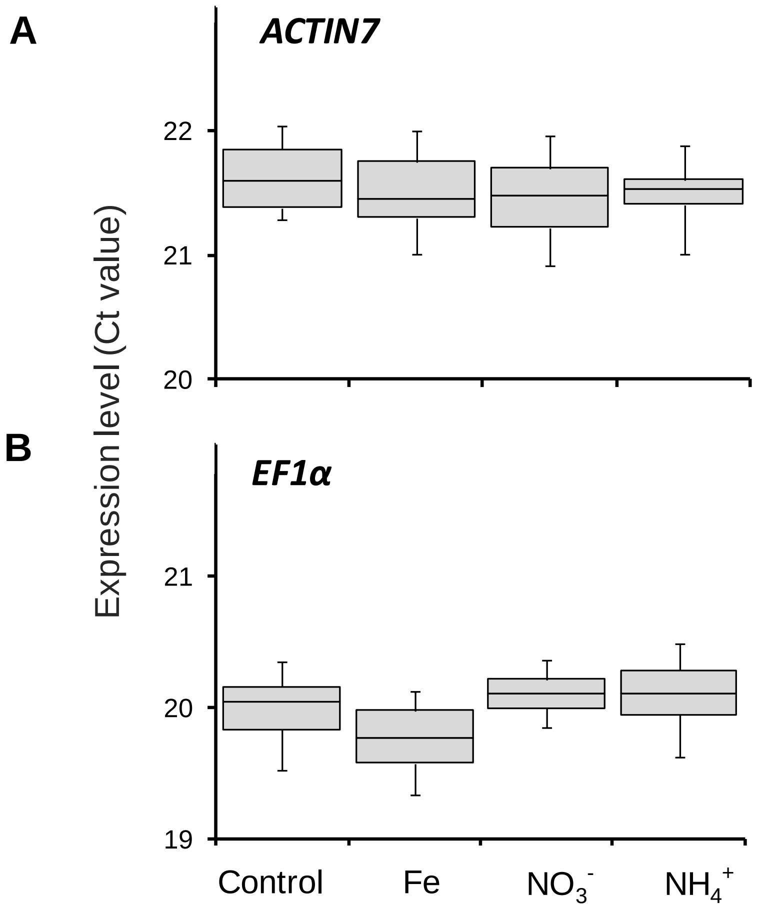

Fig. S1 RT-qPCR analysis of transcript abundance of *ACTIN7* (A) gene in comparison to *EF1α* (B) in stem bases of *Petunia hybrida* cuttings supplied with iron, ammonium or nitrate. Global expression levels (Ct values) at 0, 1 and 7 dpe are shown as 25th and 75th percentiles (top and bottom box borders); median values (middle horizontal line); minimal and maximal values (whiskers).

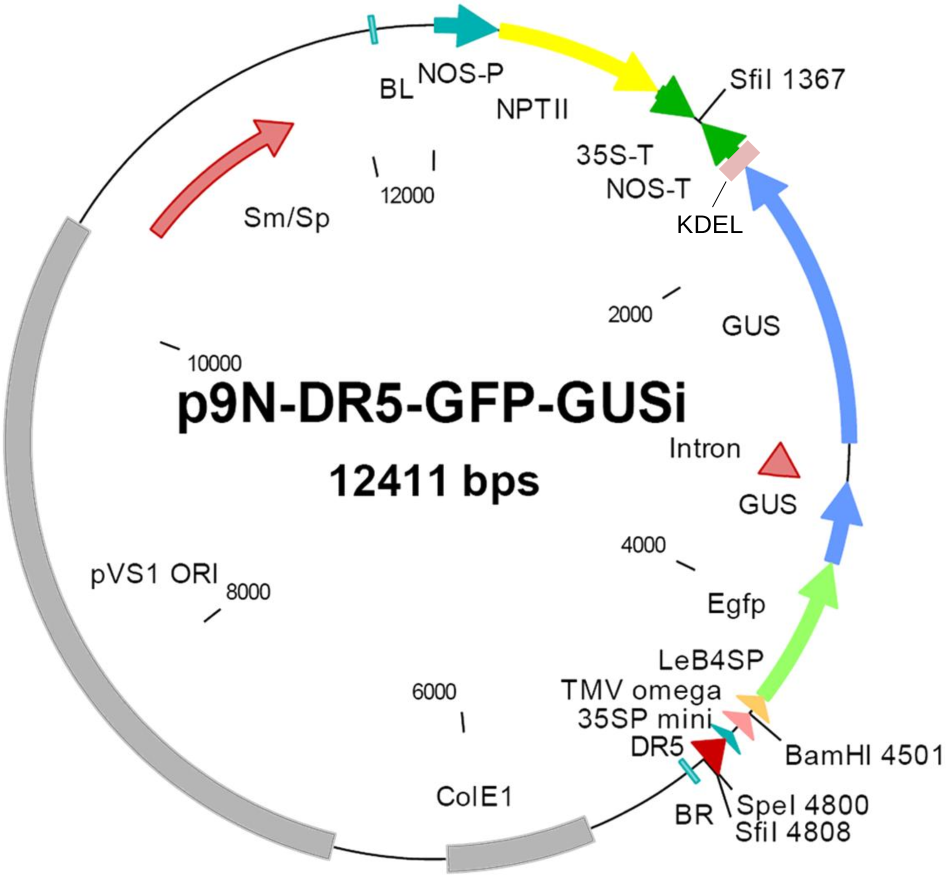

Fig. S2 Map of the binary vector p9N-DR5-GFP-GUSi, used to generate the DR5::GFP/GUS auxin-reporter line of *Petunia hybrida* cv. Mitchell. BR/BL, the left and right border sequences of T-DNA; ColE1, replication origin; DR5, synthetic auxin-responsive promoter; Egfp, enhanced green fluorescent protein; GUS, β-glucuronidase; KDEL, signal peptide for endoplasmic reticulum localization, LeB4SP, legumin B4 signal peptide for endoplasmic reticulum localization; NOS-P, promoter of a nopaline synthase gene; NOS-T, terminator of a nopaline synthase gene; NPTII, neomycin phosphotransferase gene for kanamycin resistance; pVS1 ORI, minimal replication origin of the pVS1 plasmid; Strep/Spec, gene for resistance to spectinomycin and streptomycin; TMV omega, leader sequence of tobacco mosaic virus; t-OCS, terminator of an octopine synthase gene.

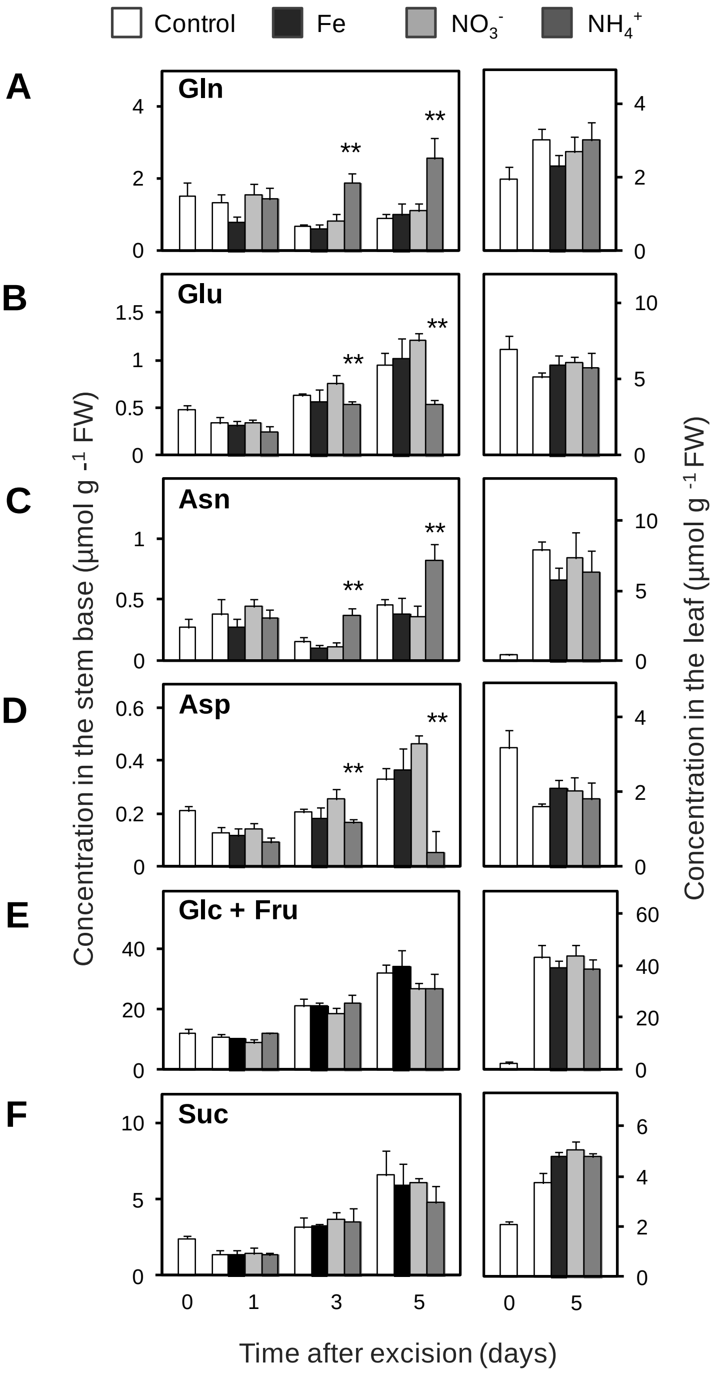

Fig. S3 Concentrations of major amino acids (A-D) and carbohydrates (E-F) in the stem base and mature leaves of *Petunia hybrida* cuttings during adventitious root formation in response to nutrient application. (A) Glutamine, (B) glutamic acid, (C) asparagine, (D) aspartic acid, (E) glucose and fructose, (F) sucrose. Bars represent means of five independent replicates + SE. Significant differences to control treatments at specified time points after excision are indicated by asterisks (t-test; *, $P < 0.05$; **, $P < 0.01$).

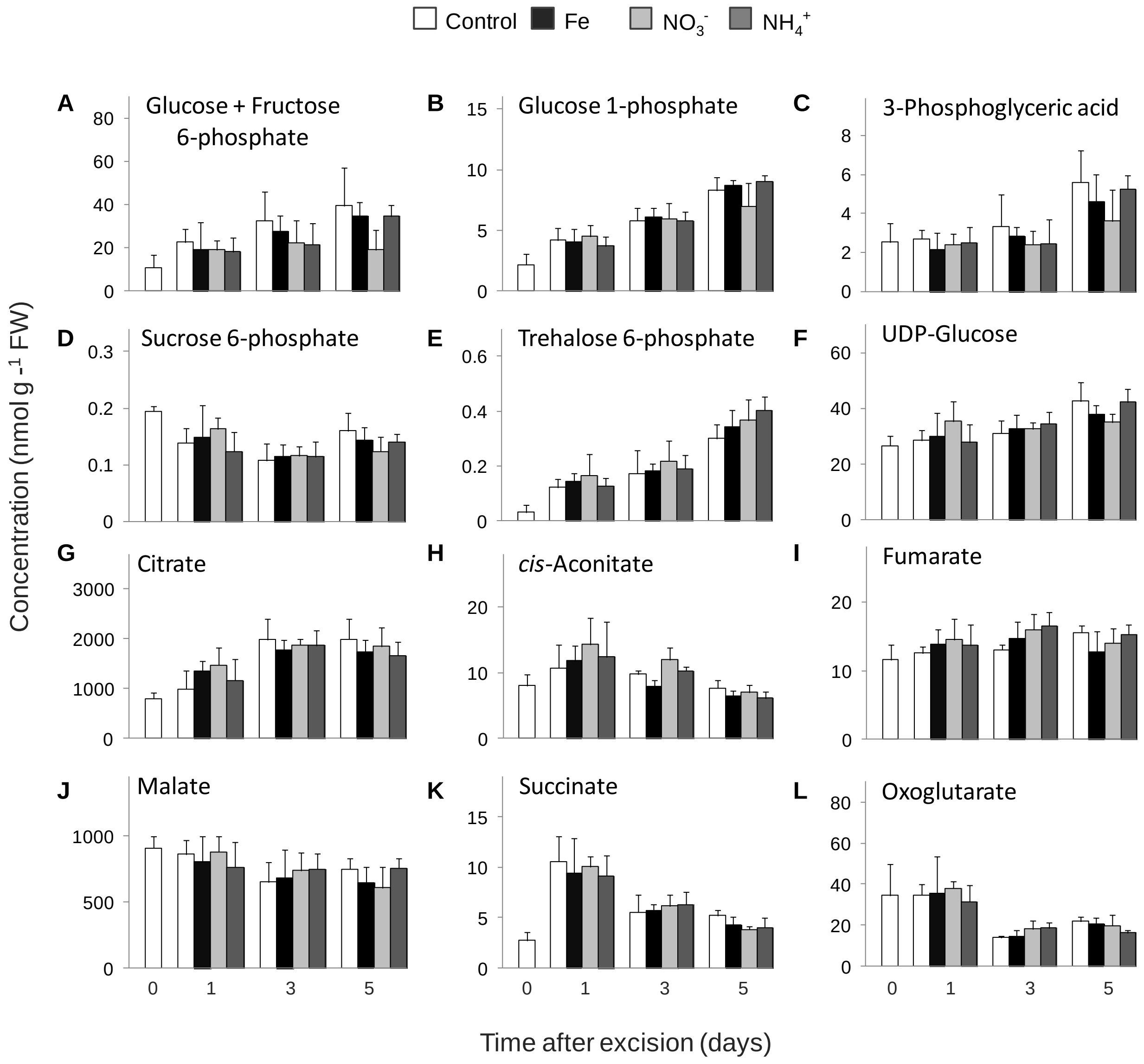

Fig. S4 Concentrations of primary intermediates of sugar metabolism in the stem base of *Petunia hybrida* cuttings during adventitious root formation in response to nutrient application. Concentrations of (A) glucose 6-phosphate and fructose 6-phosphate, (B) glucose 1-phosphate, (C) 3-phosphoglyceric acid, (D) sucrose 6-phosphate, (E) trehalose 6-phosphate, (F) UDP-glucose, (G) citrate, (H) *cis*-aconitate, (I) fumarate, (J) malate, (K) succinate and (L) oxoglutarate. Bars represent means of five independent replicates + SE.

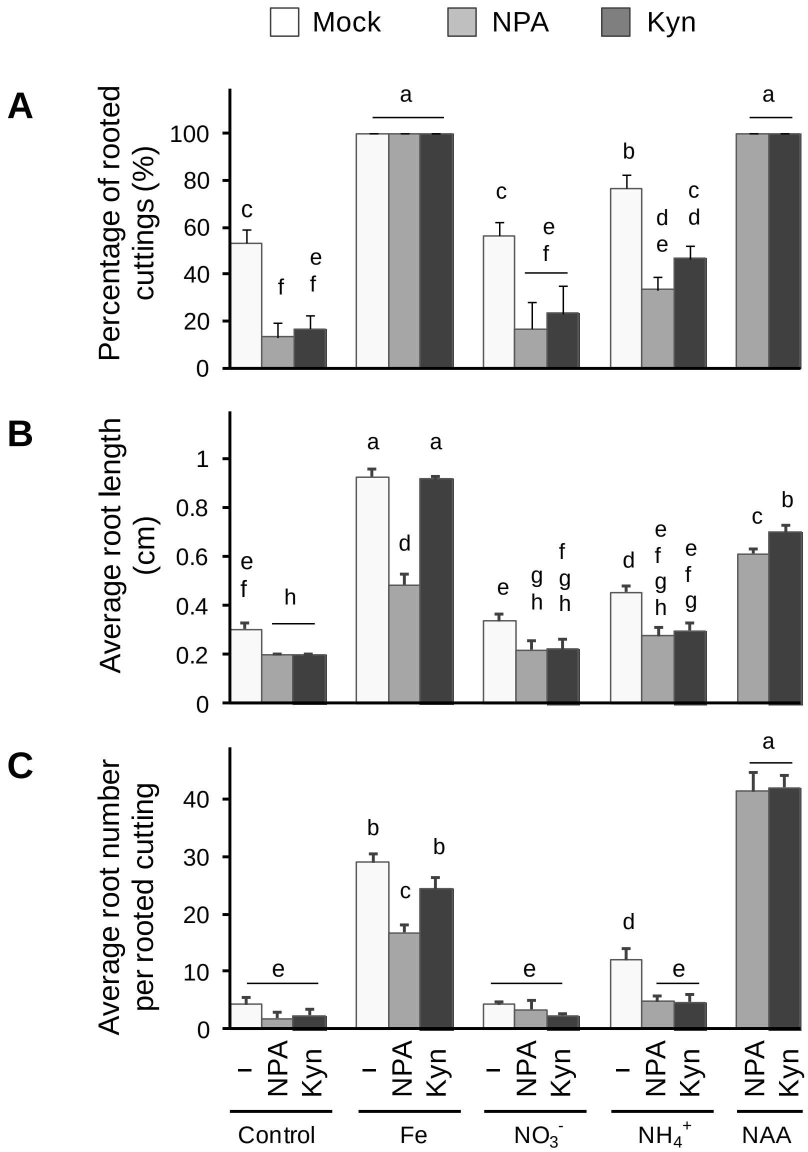

Fig. S5 Effect of auxin inhibitors on adventitious root (AR) formation in *Petunia hybrida*. Application of the auxin transport inhibitor, 80 µM naphthylphthalamic acid (NPA), or the auxin biosynthesis inhibitor, 1 µM L-kynurenine (Kyn), was combined with the application of iron, nitrate, ammonium in otherwise nutrient-free conditions (control). (A) Percentage of rooted cuttings, (B) average root length and (C) average number of ARs were assessed 14 days after excision. Combination of 3 µM 1-naphthaleneacetic acid (NAA), applied to the rooting medium during 0-2 dpe. NPA or Kyn was used to control the effect of auxin in overcoming the negative effect of studied inhibitors. Each bar represents the mean of three biological replicates, each consisting of 10 cuttings + SE. Significant differences are indicated by different letters (Tukey's HSD, P ≤ 0.05).

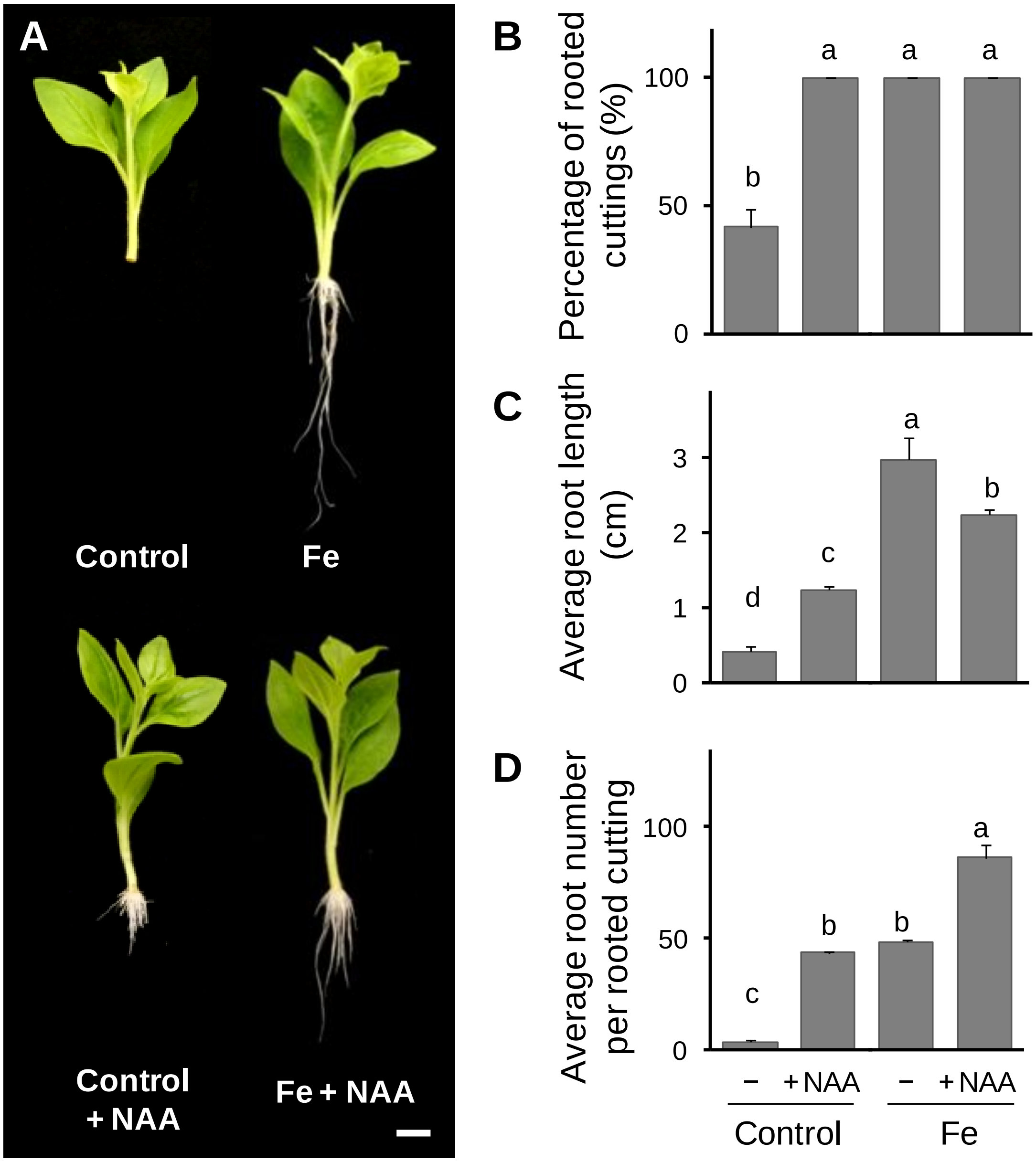

Fig. S6 Effect of the application of NAA to the cuttings of *Petunia hybrida,* supplied with Fe or grown in nutrient-free medium. 3 μM 1-naphthaleneacetic acid (NAA, Sigma-Aldrich) was applied to the rooting medium during 0-2 dpe. (A) Representative image of rooted cuttings supplied with Fe or grown in nutrient-free medium with or without NAA treatment. (B) Percentage of rooted cuttings, (C) average root length and (D) average number of adventitious roots were assessed 14 days post excision. Bars represent means of three independent replicates, each consisting of 8 cuttings + SE. Significant differences between auxin treatments and nutrient supplies are indicated by different letters (Fisher's LSD, $P \leq 0.05$). Scale bar, 1 cm.

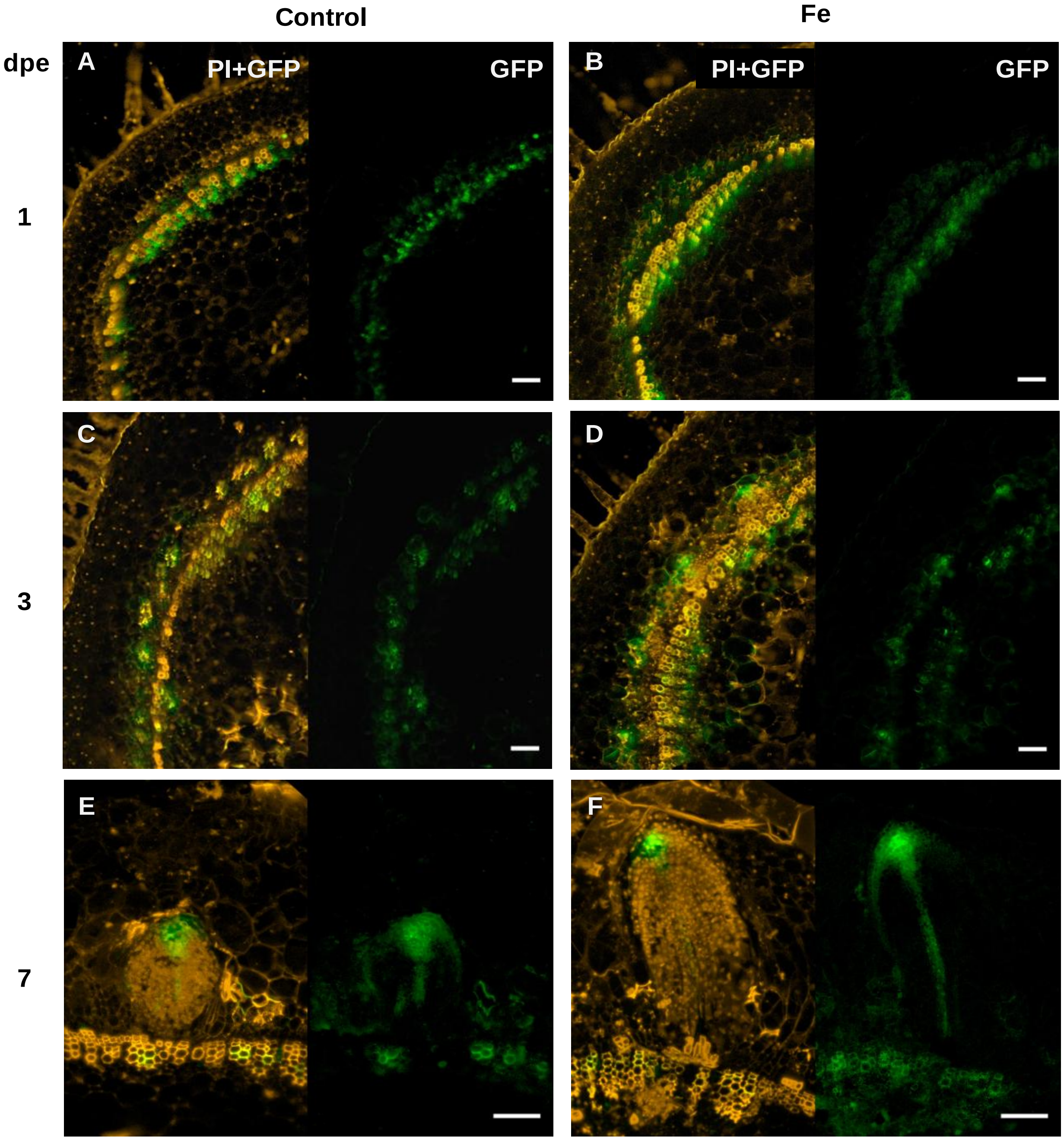

Fig. S7 Auxin-induced GFP fluorescence in the stem base of *Petunia hybrida* DR5::GUS-GFP reporter line cuttings during adventitious root formation in response to iron application. Hand-cut sections of the stem base were counterstained by 5 mg l^{-1} propidium iodide (PI, Sigma-Aldrich). Fluorescence of GFP was probed with a 488-nm laser line (2.5 % intensity) and recorded between 491-535 nm. Combined fluorescence of PI and GFP was visualized by a 488-nm laser line (2.5 % intensity) over the range 491-597 nm. Representative sections are shown for 1 dpe (A, B), 3 dpe (C, D) or 7 dpe (E, F) in iron-supplied cuttings (B, D, F) compared to control conditions without nutrients (A, C, E). Scale bar, 100 µm.

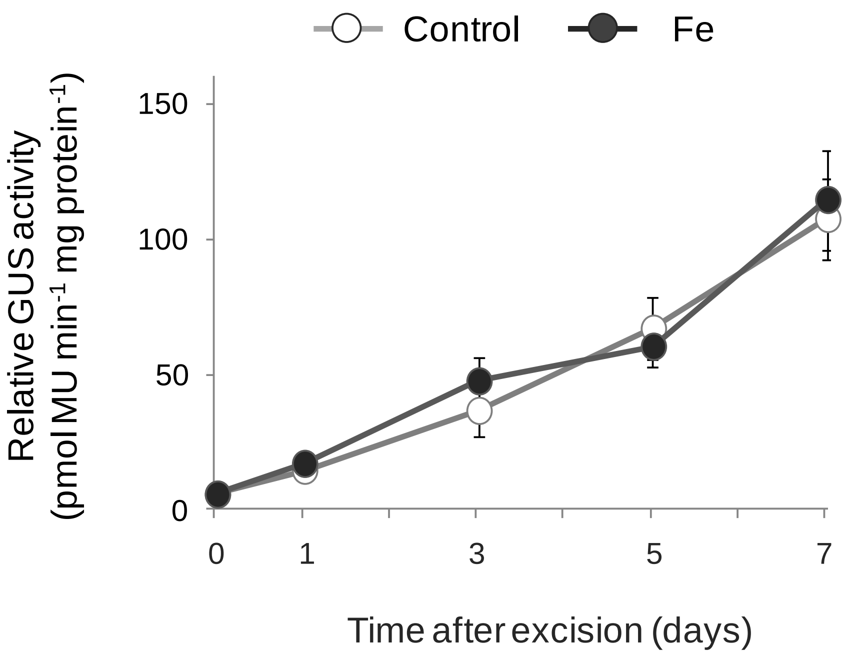

Fig. S8 Analysis of β-glucuronidase (GUS) activity in the stem base of a *Petunia hybrida* DR5::GUS-GFP reporter line cuttings during adventitious root formation in response to nutrient application. The GUS-activity was analyzed in a fluorometric assay according to Jefferson *et al.* (1987) using 4-methylumbelliferyl β-D-glucuronide as a substrate for GUS. Fluorescence was recorded with a microplate reader (Infinite 200, Tecan) at 460 nm when excited at 355 nm. Protein concentrations were determined using Bradford's protein assay kit (Bio-Rad), and final GUS activity was expressed as pmol 4-methylumblliferone (MU) min^{-1} mg $protein^{-1}$. Each data point represents the mean of five independent replicates ± SD from one transgenic line.

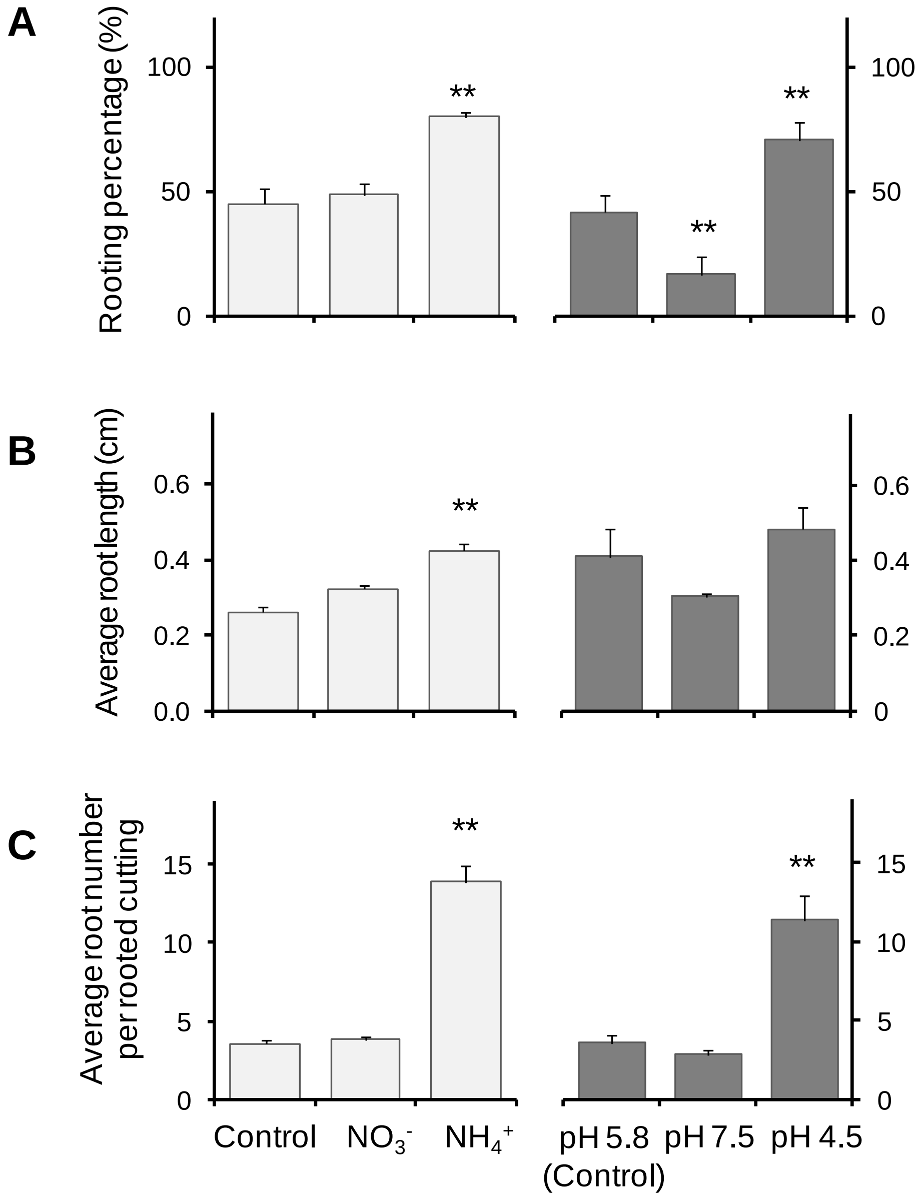

Fig. S9 Effect of the medium pH on adventitious root (AR) formation in *Petunia hybrida* in comparison to the average rooting performance under ammonium and nitrate supply. (A) Percentage of rooted cuttings, (B) average root length and (C) average number of AR were assessed 14 days after excision. For different pH conditions bars represent means of three independent replicates, each consisting of 8 cuttings + SE. For average ammonium and nitrate supply bars represent means of four independent experiments + SE. Significant differences to control treatments are indicated by asterisks (t-test; *, $P < 0.05$; **, $P < 0.01$).

Table S1 Protocol for microwave-assisted fixation, dehydration and resin embedding of *Petunia hybrida* cutting base samples for histological analysis

Process	Reagent	Power, W	Time, sec	Vacuum, mmHg
1. Primary fixation	2 % (v/v) glutaraldehyde and	150	60	15
	2 % (w/v) paraformaldehyde	0	60	15
	in 0.05 M cacodylate buffer (pH 7.3)	150	60	15
		0	60	15
		150	60	15
	Followed by incubation for 16h on a rotary shaker at RT			
2. Washing	- 0.05 M cacodylate buffer (pH 7.3)	150	45	0
	- bidistilled water, repeated two times	150	45	0
3. Secondary fixation	1% (w/v) OsO_4	0	60	0
		80	120	15
		0	60	0
		80	120	15
	Followed by incubation for 45 min on a rotary shaker at RT			
4. Washing	bidistilled water, repeated three times	150	45	0
5. Dehydration	- 100 % acetone	150	45	0
	- 30 %, 40 %, 50 %, 60 %, 70 %, 80 %, 90 % acetone in propylenoxide	150	45	0
	- 100 % propylenoxide	150	45	0
	Each step followed by incubation for 20 min on a rotary shaker at RT			
6. Resin infiltration	Spurr´s resin in propylenoxide:	Incubation on rotary shaker at RT:		
	- 20 %		12 h	
	- 40 %, 60 %, 80 %		3h, each	
	- 100 % Spurr´s resin		12 h	
7. Polymerization	Transfer into BEEM capsules with fresh Spurr´s resin		24 h, at 70°C	

Table S2 Protocol for preparation of histological samples of the stem base segments of *Petunia hybrida* cuttings

Process	Reagent	Conditions
1. Fixation	- 4% (v/v) glutaraldehyde, 1% (v/v) paraformaldehyde in 0.25 M phosphate buffer (pH 7.3)	48 h at 4°C
2. Washing	bidistilled water, repeated two times	1 min at RT
3. Vibratome sectioning	- embedding of stem samples in 4% agarose<br>- serial transverse sectioning with 1mm interval and thickness of 100 µm	
4. Staining	- 1% (w/v) methylene blue and 1% (w/v) azure II in 1% (w/v) aqueous borax solution	2 min at RT
5. Washing	bidistilled water, repeated two times	1 min at RT

Table S3 Settings for MS/MS analysis of primary metabolites

Compound	Transition (m/z) precursor ion → product ion	Dwell time, sec	Collision energy, eV	Polarity
cis-Aconitate	173.1 → 85	20	9	-
	173.1 → 128.9	20	1	-
Citrate	191 → 86.8	20	13	-
	191 → 110.8	20	15	-
Fructose 6-phosphate	259 → 78.9	20	44	-
	259 → 96.8	20	9	-
Fumarate	115 → 70.9	20	1	-
Glucose 1-phosphate	259 → 78.9	20	44	-
	259 → 96.8	20	9	-
Glucose 6-phosphate	259 → 78.9	20	44	-
	259 → 96.8	20	9	-
Malate	133 → 71	20	9	-
	133 → 115	20	5	-
Oxoglutarate	145.1 → 101	20	5	-
3-Phosphoglyceric acid	185.1 → 78.8	20	41	-
	185.1 → 96.8	20	20	-
Succinate	116.9 → 73	20	5	-
Sucrose 6-phosphate	421 → 79.1	20	53	-
	421 → 96.8	20	33	-
Trehalose 6-phosphate	421 → 139	20	29	-
	421 → 240.9	20	25	-
UDP-Glucose	565 → 158.9	20	53	-
	565 → 322.9	20	25	-

Table S4 Primers used to study the transcript abundance of marker genes for cell division, nutrient acquisition and auxin homeostasis in *Petunia hybrida*

Gene	Forward primer (5'-3')	Reverse primer (5'-3')
ACTIN7	TCAGATTTGCTGGCATGAAG	ATTGTCCAAAGCAAGGATGG
AMT1	TGCTAAAGGGAGCTATGTGGA	TGGATTATATGTGCCCCAAG
CYCLINB1	GGTTACACGTCGTGGTGTTG	TCTGAGCTGCAGGTTTCCTT
CYCLIN2	ATGTCGAGGAACATGAGGCA	TGGATTCTTTGCATCATCACCA
EF1α	CCTGGTCAAATTGGAAACGG	GATCGCCTGTCAATCTTGG
IRT1	TTGCTCAATGCATCTTCTGC	GGACATTCCACCAGCACCTA
FERRITIN	CAGAACAAGCGTGGTGGAAA	ACGTCGTTGTTTTCTGAGGC
FRO2	TGTGTGGAAGCAGGTCCATA	TTCAAAATTGCAATGGCATC
NAS	TGGCTCTTATGAAAACCCACT	GGAGAGGGCCTGATCCAATA

Table S5 Effect of different concentrations of Fe and of N supply on adventitious root (AR) formation in *Petunia hybrida* cuttings

**Rooting parameters**[1]	**Fe EDTA**, µM				NH_4NO_3, mM			
	4	8	12	16	0.1	0.2	0.5	1
Rooting percentage	1.6±0	1.6±0	1.7±0.0*	1.7±0.0*	1.1±0.0	1.7±0.2*	1.5±0.1*	1.5±0.1*
Average AR number per rooted cutting	1.5±0.3	2.1±0.7*	7.1±1.0**	6.0±0.9**	1.2±0.2	2.9±0.2**	2.5±0.3*	2.7±0.3*
Average AR length	1.4±0.1**	3.4±0.2**	9.3±1.3**	9.0±1.4**	1.1±0.2	1.4±0.1*	1.3±0.1	1.3±0.0

[1] Values represent an increase in rooting parameters under nutrient application in relation to the control conditions. Each value represents the mean of three independent replicates consisting of 8 cuttings ± SE. Significant differences to control treatments are indicated by asterisks (t-test; *, $P < 0.05$; **, $P < 0.01$)
